# ALKBH5 inhibitors for cancer therapy: molecular docking, challenges and future prospects

**DOI:** 10.3389/fphar.2026.1946973

**Published:** 2026-09-25

**Authors:** Chen Deng, Hai Xu, Jian Pu, Huamei Wei, Wenjin Chen, Yubin Luo, Shengxiao Zhang, Haifeng Jin, Jie Liao, Zhongpeng Fan, Miaomiao Cao, Yangyang Xu, Benshun Hu

**Affiliations:** 1 Norman Bethune International Institute of Canada for Healthcare Innovation, Toronto, ON, Canada; 2 International Institute of Synthetic Biology - Canada, Toronto, ON, Canada; 3 Guangxi Clinical Medical Research Center of Hepatobiliary Disease, Baise, China; 4 Department of Gynecology, Hubei Shizhen Laboratory, Huangjiahu Hospital of Hubei University of Chinese Medicine, Hubei University of Chinese Medicine, Wuhan, China; 5 Department of Hepatobiliary Surgery, Affiliated Hospital of Youjiang Medical University for Nationalities, Baise, China; 6 Department of Pathology, Affiliated Hospital of Youjiang Medical University for Nationalities, Baise, China; 7 Department of Urology, Xinhua Hospital, Shanghai Jiao Tong University School of Medicine, Shanghai, China; 8 Department of Rheumatology and Immunology, West China Hospital, Sichuan University, Chengdu, China; 9 Department of Rheumatology and Immunology, The Second Hospital of Shanxi Medical University, Taiyuan, Shanxi, China; 10 Department of Gastroenterology and Gastrointestinal Endoscopy Center, Bethune International Peace Hospital, Shijiazhuang, China; 11 Department of Hepatobiliary Surgery, Affiliated Hospital of Jiangnan University, Wuxi, China

**Keywords:** ALKBH5, cancer therapy, challenges, future prospects, RNA demethylase

## Abstract

N^6^-Methyladenosine (m^6^A), the most abundant internal modification of eukaryotic mRNA, is dynamically reversed by the Fe^II^/α-ketoglutarate-dependent dioxygenase ALKBH5, a key m^6^A “eraser” that regulates target mRNA fate through demethylation. ALKBH5 is aberrantly overexpressed in acute myeloid leukemia, glioblastoma, breast cancer and other malignancies, where it sustains oncogenic programs, establishing it as a promising epitranscriptomic drug target. At present, ALKBH5 inhibitor development lags behind that of FTO, with selective agents only recently emerging through covalent inhibitors such as compound **33**, **25** and **40**, though no clinically available ALKBH5-targeted drug yet exists. This review summarizes the biological functions, structural features, and molecular docking between reported inhibitors and target proteins of ALKBH5. Current challenges, including selectivity over homologous dioxygenases, structural optimization and resistance mechanisms, are critically discussed, alongside future perspectives on structure-guided design, combination regimens and clinical translation. This review provides a medicinal chemistry framework to accelerate the discovery of potent and selective ALKBH5 inhibitors for precision cancer therapy.

## 1 Introduction

Among the diverse post-transcriptional modifications occurring in eukaryotic messenger RNA, N^6^-methyladenosine (m^6^A) stands out as the most frequently observed internal epigenetic mark, exerting profound influence over the control of gene expression programs (Vasilopoulou et al., 2025). This reversible RNA modification cycle depends on the coordinated action of writers, erasers, and readers. Methyl group deposition is catalyzed by complexes containing methyltransferase-like 3 and 14 (METTL3/14) heterodimers along with regulatory partners Wilms tumor 1-associated protein (WTAP) and RNA-binding motif protein 15 (RBM15). Conversely, ALKBH5 reverse this process via demethylation. The functional output of m^6^A marking is then interpreted by YTH domain family (YTHDF), YTH domain-containing (YTHDC), and insulin-like growth factor 2 mRNA-binding protein (IGF2BP) family proteins, whose docking at modified adenosines redirects the post-transcriptional trajectory of target transcripts through altered splicing, export, translation efficiency, and stability (Wu et al., 2025). Through the precise cooperation of these three classes of proteins, as a pervasive epitranscriptomic mark, m^6^A methylation underpins an elaborate post-transcriptional regulatory architecture that modulates diverse biological programs, spanning from the arbitration of cell fate decisions and the preservation of stem cell pluripotency to developmental patterning, lineage commitment, and neoplastic transformation. Contemporary investigations have increasingly established that quantitative and qualitative perturbations in m^6^A deposition, alongside dysfunctional operation of its cognate regulatory machinery, serve as critical pathogenic determinants underlying a wide spectrum of human disorders, with oncogenesis and malignant progression representing the most extensively characterized manifestations (Wu et al., 2025).

ALKBH5, as an important member of the AlkB family, serves as a key “eraser” within the m^6^A modification system, removing m^6^A modifications from mRNAs through its demethylase activity, thereby regulating the expression of target genes. Numerous studies have shown that ALKBH5 is aberrantly overexpressed in various cancers. In acute myeloid leukemia (AML), Shen et al. discovered that ALKBH5, contrary to previously reported frequent deletion in human AML, is actually abnormally highly expressed, and its elevated expression levels are significantly correlated with poor prognosis in AML patients (Shen et al., 2020). That investigation additionally established that ALKBH5 constitutes an indispensable factor for both leukemogenesis and sustained disease progression, as well as for the propagation of leukemia stem/initiating cells, yet proves non-essential for physiological hematopoietic function (Shen et al., 2020). Mechanistic investigations revealed that ALKBH5 post-transcriptionally regulates key target genes, such as the oncogene transforming acidic coiled-coil-containing protein 3 (TACC3) and the receptor tyrosine kinase AXL by demethylating their mRNAs, enhancing their stability and promoting their expression, thereby driving tumor stem cell self-renewal and malignant proliferation of cancer cells (Shen et al., 2020). Moreover, ALKBH5 has been shown to participate in AML progression and immune evasion through the regulation of additional downstream targets, including inosine triphosphatase (ITPA) and programmed death-ligand 1 (PD-L1) (Liu B et al., 2020; Ma et al., 2026). Anomalous ALKBH5 overexpression extends beyond myeloid leukemia into a range of epithelial-derived cancers. In breast and lung malignancies, for instance, this eraser enzyme enhances metastatic spread and local tissue invasion by operating through RNA modification-dependent processes akin to those characterized in AML (Petri and Klinge, 2023). These findings collectively establish ALKBH5 as a core oncogenic factor and position it as a highly promising therapeutic target for cancer.

Relative to the comparatively mature therapeutic landscape of FTO inhibitors, the discovery and optimization of robust, ALKBH5-selective small-molecule antagonists remain substantially underdeveloped. FTO, as the first identified m^6^A demethylase, has been targeted by multiple inhibitors that have shown potential in cancer therapy. For example, loratadine, discovered through a drug repurposing strategy, exhibits FTO inhibitory activity in esophageal cancer (Mohammadi et al., 2025), and FTO inhibitors have also demonstrated application prospects in overcoming osimertinib resistance in lung adenocarcinoma (Chen et al., 2025). However, reports on inhibitors specifically targeting ALKBH5 are still limited. Most currently reported ALKBH5 inhibitors are either 2-oxoglutarate (2-OG) analogs or pan-inhibitors that simultaneously target both FTO and ALKBH5, and truly highly selective ALKBH5 inhibitors remain extremely scarce. Recently, the team of Liu Hong and Yang Caiguang at the Shanghai Institute of Materia Medica reported a covalent inhibitor 18 targeting Cys200 of ALKBH5, which exhibits potent ALKBH5 inhibitory activity and favorable selectivity, demonstrating excellent anti-tumor effects in both *in vitro* and *in vivo* AML models (Wu et al., 2025). Additionally, Fei et al. reported the salicylaldehyde-based covalent inhibitor compound 25 (Fei et al., 2025), and Wang and colleagues identified a compound 40 as an ALKBH5 inhibitor (Liang et al., 2025). These breakthrough advances indicate that the development of selective ALKBH5 inhibitors is accelerating. Nevertheless, clinically available ALKBH5-targeted drugs are still lacking. The discovery of highly potent and selective ALKBH5 inhibitors is expected not only to provide a novel targeted therapeutic strategy for cancers with overexpression of m^6^A demethylases but also to serve as indispensable chemical probe tools for in-depth dissection of the biological functions of ALKBH5 in tumorigenesis.

## 2 Biological functions of ALKBH5

Functioning as an Fe^II^/α-ketoglutarate (α-KG, also known as 2-oxoglutarate, 2-OG)-dependent oxidative demethylase, ALKBH5 selectively erases m6A epigenetic marks from single-stranded RNA (ssRNA) molecules. This catalytic activity is mediated through its evolutionarily preserved double-stranded β-helix (DSBH) core architecture, complemented by specialized recognition loops that mediate precise engagement with target ribonucleotides (Aik et al., 2014; Zheng et al., 2013). Physiologically, it is indispensable for male spermatogenesis and oocyte meiotic maturation by controlling m^6^A-dependent mRNA splicing and stability (Bai et al., 2023; Tang et al., 2018). In the nervous system, ALKBH5 regulates synaptic plasticity and axon regeneration, whereas its dysregulation exacerbates cerebral ischemia reperfusion injury (Qiu et al., 2024; Wang D. et al., 2023). Cardioprotective roles include promoting cardiomyocyte proliferation and heart regeneration through YTHDF1 demethylation (Han et al., 2021), conversely, its upregulation in failing hearts and acute myocardial infarction correlates with metabolic reprogramming and aggravated injury (Cheng et al., 2022; Komal et al., 2023). Additionally, hepatic ALKBH5 governs glucose and lipid homeostasis through GCGR and mTORC1 signaling (Ding et al., 2023), and in the immune system, it modulates CD4^+^ T cell pathogenicity, γδ T cell development, and neutrophil antibacterial migration (Ding et al., 2022; Liu Y. et al., 2022; Zhou et al., 2021).

Eukaryotic messenger RNAs harbor numerous post-transcriptional modifications, yet none is more abundant than the internal N^6^-methyladenosine (m^6^A) residue. This reversible mark is established through the coordinated activity of writer methyltransferases, interpreted by reader proteins that recognize the modification, and reversed by eraser demethylases—three distinct but interdependent enzymatic classes (Gao et al., 2024; Qu et al., 2022). The methyltransferase complex (MTC) drives m^6^A installation through a bipartite organizational architecture, the METTL3-METTL14 module provides the catalytic activity, while auxiliary subunits—WTAP, VIRMA, RBM15, and ZC3H13—coordinate RNA substrate engagement and complex scaffolding (Qu et al., 2022). Once deposited, the m6A epigenetic tag is preferentially decoded by a dedicated class of reader effectors, notably the YTH-domain-containing protein family comprising the cytoplasmic paralogs YTHDF1, YTHDF2, and YTHDF3 alongside the nuclear isoforms YTHDC1 and YTHDC2 and the IGF2BP family, which direct the modified transcripts toward alternative splicing, nuclear export, enhanced translation, or regulated decay (Gao et al., 2024; Qu et al., 2022). As shown in Figure 1, the erasure of m^6^A is executed by two Fe^II^/*α*-ketoglutarate-dependent dioxygenases, and ALKBH5, which oxidatively remove the methyl group to regenerate unmodified adenosine, thereby resetting the epitranscriptomic state and allowing dynamic adaptation to cellular and environmental cues (Gao et al., 2024; Qu et al., 2022). Disruption of this finely tuned circuitry, through aberrant expression or altered activity of writers, readers, or erasers has been increasingly recognized as a fundamental mechanism underlying malignant transformation, metabolic disorders, and immune dysregulation (Qu et al., 2022).

**FIGURE 1:**
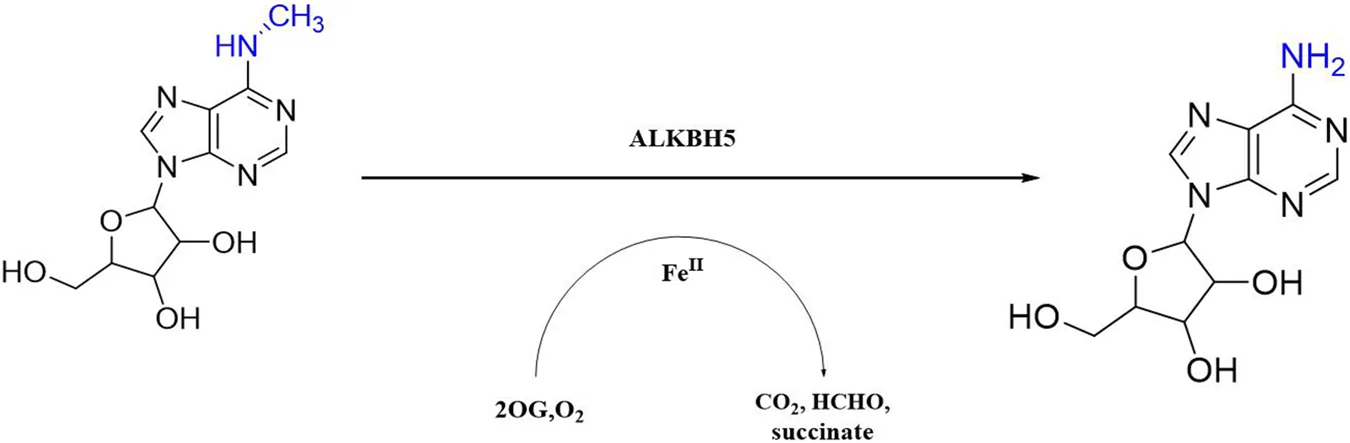
Distinct oxidative demethylation mechanisms of ALKBH5. ALKBH5 catalyzes the direct, single-step conversion of N^6^-methyladenosine (m^6^A) to adenosine (A). Both enzymatic reactions are strictly dependent on Fe^II^ and α-ketoglutarate (α-KG) as essential cofactors for dioxygenase activity.

From an oncological perspective, ALKBH5 assumes a pro-tumorigenic identity in a subset of aggressive cancers. Specifically, in acute myeloid leukemia, glioblastoma, and breast cancer, the enzyme fuels malignant transformation and disease maintenance by actively demethylating key oncogenic messages—namely TACC3, AXL, forkhead box M1 (FOXM1), and homeobox transcription factor NANOG (NANOG)—thereby protecting these transcripts from degradation and ensuring the perpetual renewal and rapid division of the cancer stem cell pool (Li, R. et al., 2023; Shen et al., 2020; Zhang et al., 2016; Zhang et al., 2017). Conversely, it acts as a tumor suppressor in hepatocellular carcinoma (HCC) and pancreatic cancer by destabilizing pro-tumorigenic mRNAs such as *LYPD1* or activating *PER1* and *WIF1* to suppress malignant progression (Chen et al., 2020; Guo et al., 2020). Accumulating data further establish ALKBH5 as a dominant modulator of ferroptosis. Within hypopharyngeal squamous carcinoma and thyroid cancer contexts, the enzyme fosters this regulated necrotic process through the transcriptional or post-transcriptional inhibition of the Nrf2/heme oxygenase-1/glutathione peroxidase 4 defensive axis, yet inhibits ferroptosis in hepatocellular carcinoma and glioblastoma stem cells via non-canonical protein interactions [e.g., circRNA cIARS-mediated BCL-2/BECN1 stabilization (Liu Z. et al., 2020)] or EGFR/SRC-driven nuclear retention that upregulates GCLM and glutathione synthesis (Li, W. et al., 2023; Liu L et al., 2024; Lv et al., 2023; Zhuang et al., 2025). These divergent functions are further fine-tuned by post-translational modifications, including ROS-induced SUMOylation, KAT8-mediated acetylation, USP36-mediated deubiquitination, and EGFR-activated phosphorylation, which collectively modulate ALKBH5 enzymatic activity, subcellular localization, and substrate specificity (Chang et al., 2023; Lv et al., 2023; Yu F. et al., 2021; Zhang et al., 2023).

## 3 ALKBH5 structure

Human ALKBH5 is a 394-residue non-heme Fe^II^/*α*-ketoglutarate-dependent dioxygenase belonging to the ALKB superfamily (Trewick et al., 2002; Zheng et al., 2013). In Figure 2a, its primary structure is organized into three functional segments: an *N*-terminal alanine-rich region containing a coiled-coil motif that is implicated in nuclear speckle localization (Fu et al., 2014), a central catalytic core (residues 66–292) harboring the canonical Fe^II^-binding HXDXnH motif (His204-Asp206-His266) and the RXnR signature for *α*-ketoglutarate (2OG) coordination (Aik et al., 2014; Feng et al., 2014), and a C-terminal intrinsically disordered extension enriched in arginine-serine repeats that likely modulates RNA substrate engagement (Aik et al., 2014).

**FIGURE 2:**
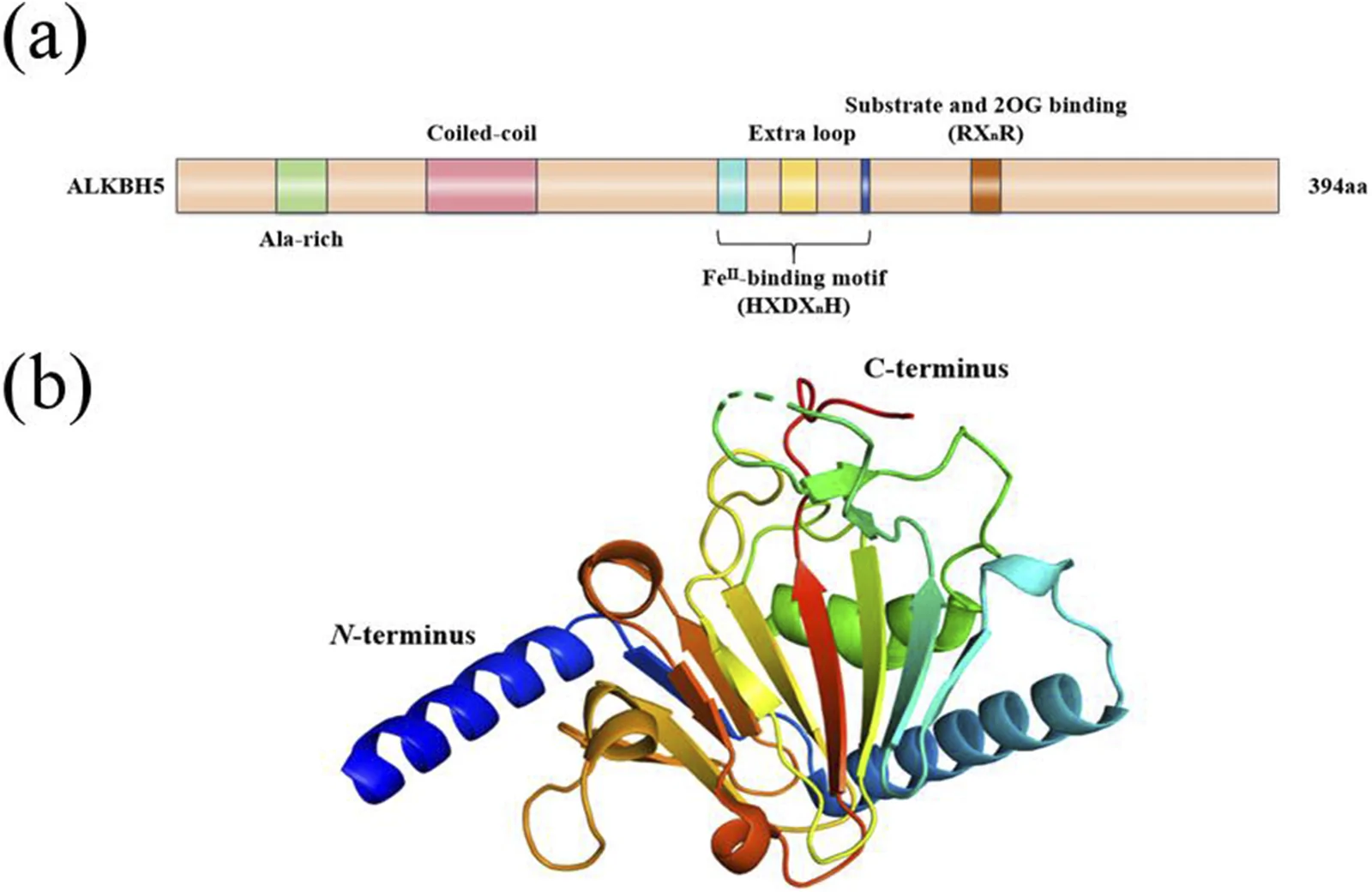
**(a)** The 2D structure of ALKBH5. Linear schematic showing the Ala-rich (yellow), Coiled-coil (green), FeII-binding motif (HXDXnH) (blue, gray, dark blue), Substrate and 2OG binding (dark red). **(b)** Crystal structure of human ALKBH5 (PDB: 4NJ4). The protein is displayed in cartoon representation with rainbow spectrum coloring from the N-terminus (blue) to the C-terminus (red).

Figure 2b presents the crystallographic structure of the catalytic domain, revealing a conserved DSBH motif where strands *β*6, *β*8, *β*11, and *β*13 constitute the primary *β*-sheet, while *β*7, *β*9, *β*10, and *β*12 form the secondary counterpart; together these elements provide the active site scaffold (Aik et al., 2014). Within the active site, the catalytic metal center is ligated by a three-residue motif comprising His204, Asp206, and His266. Concurrently, α-ketoglutarate (α-KG, 2-oxoglutarate, 2-OG) within a pocket bounded by the dual *β*-sheet assembly, while the wider aperture of this groove permits the ingress of single-stranded RNA (ssRNA) substrates (Aik et al., 2014; Feng et al., 2014). The m^6^A base is recognized within a pocket comprising Arg130, Tyr139, and His204 (Feng et al., 2014). Three distinctive architectural elements enforce strict selectivity for ssRNA over double-stranded DNA or RNA: (i) the *β*9-10 extra loop (residues 229–243), which generates steric hindrance against duplex nucleic acids (Aik et al., 2014; Feng et al., 2014), (ii) an unanticipated Cys230-Cys267 disulfide bond that further occludes the active site from dsDNA/dsRNA access (Feng et al., 2014), and (iii) the nucleotide recognition lid (NRL), subdivided into NRL1 (*β*2-*β*3) and NRL2 (*β*4-*β*5), which plays a critical role in substrate orientation and catalytic fidelity (Aik et al., 2014; Feng et al., 2014). Structural comparison reveals that the catalytic chamber of ALKBH5 is considerably more compact than that of FTO. This size differential provides an exploitable structural determinant for achieving selectivity: orthosteric ligands can be tailored to sterically complement the narrower ALKBH5 cavity while clashing with the more expansive FTO active site, thereby discriminating between their respective cofactor and substrate binding domains.

## 4 The effect of ALKBH5 in malignant tumors

Functioning as a versatile RNA demethylase, ALKBH5 participates in the pathophysiology of numerous human malignancies and non-malignant conditions alike. Figure 3 highlights its tumor-specific regulatory portfolio: in breast cancer, ovarian carcinoma, glioblastoma, lung cancer, gastrointestinal neoplasms, pancreatic cancer and osteosarcoma, ALKBH5 exerts context-dependent oncogenic or tumor-suppressive functions through the epitranscriptomic control of gene products. Via these downstream effectors, ALKBH5 governs a spectrum of cellular processes encompassing cancer stem cell maintenance, autophagic flux, hypoxic adaptation, and long non-coding RNA (lncRNA) biology, consequently exerting either tumor-suppressive or tumor-promoting effects. Beyond oncology, this demethylase also contributes significantly to pathological conditions such as reproductive dysfunction and aberrant bone mineralization, operating through the perturbation of key signaling cascades including the AKT and NF-κB axes.

**FIGURE 3:**
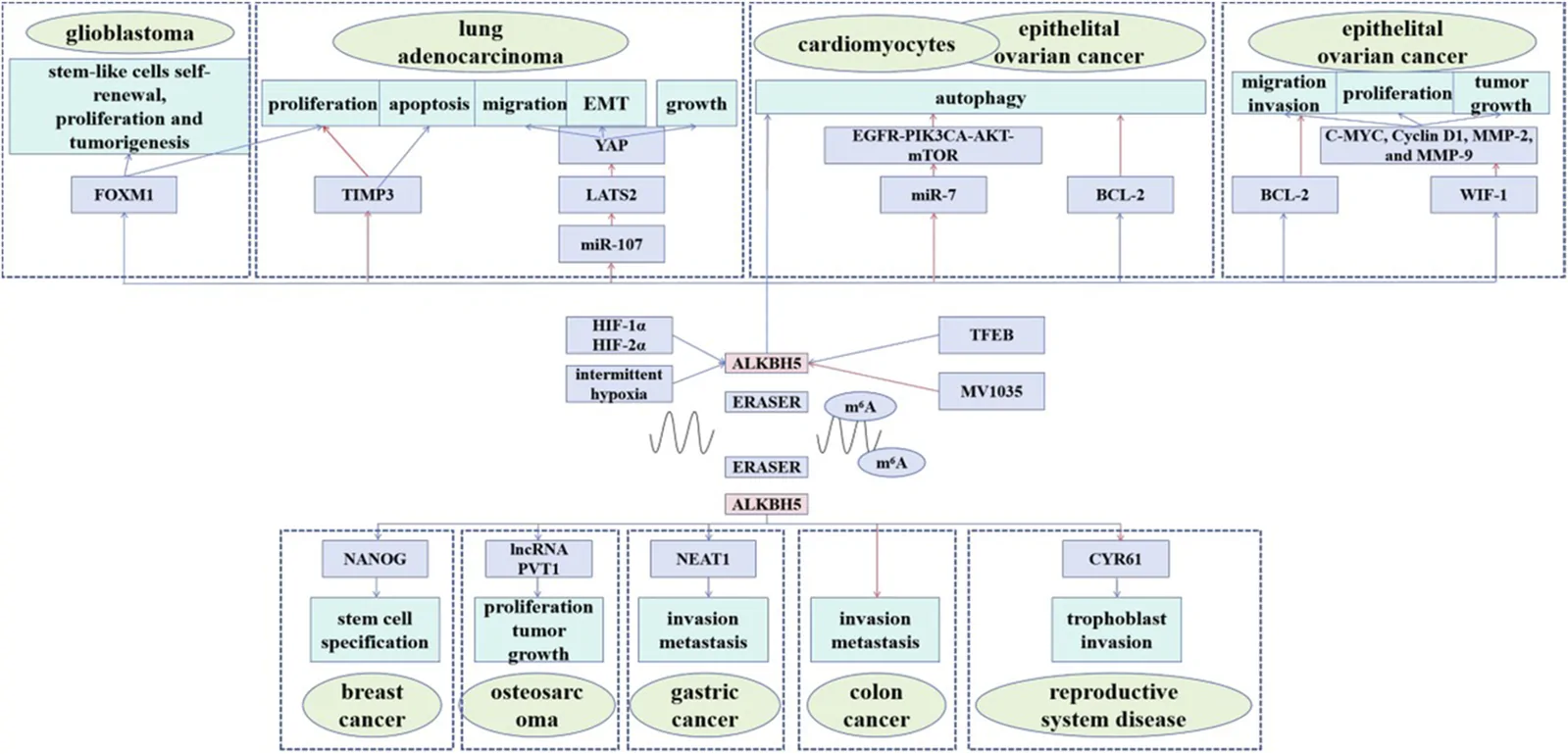
ALKBH5 driven molecular pathways are implicated in the progression of both malignant and non-malignant human diseases.

By actively stripping m6A modifications from pivotal mRNA substrates, ALKBH5 functions as a principal facilitator of carcinogenesis across multiple solid tumor types, fostering both malignant transformation and sustained neoplastic growth. However, this enzyme acts as both an oncogenic driver in certain malignancies and a tumor suppressor in specific malignant tumors, inhibiting cell proliferation and invasive potential. This functional dichotomy is increasingly attributed to the context-dependent substrate repertoire and spatiotemporal regulatory networks of ALKBH5 (Table 1).

**TABLE 1:** Effect of ALKBH5 in malignant tumors.

Role	Cancer type	Downstream targets	Molecular mechanisms	Effect	References
Tumor-suppressive role	Non-Small Cell Lung	CEMIP	Decrease mRNA stability	Inhibit cell proliferation and enhance sensitivity to chemotherapy with paclitaxel	(Gao et al., 2024)
Tumor-suppressive role	Pancreatic Cancer	WIF-1	Promote transcription	Inhibit cell proliferation, colony formation, and migration and promote chemosensitivity to gemcitabine	(Tang et al., 2020)
Tumor-suppressive role	Colorectal Cancer	*circAFF2*	-	Increase tumor radiosensitivity	(Shao et al., 2023)
Tumor-suppressive role	Hypopharyngeal Squamous Cell Carcinoma	TLR2	Enhance mRNA stability and promote translation	Inhibit growth, proliferation and promote apoptosis	(Ye et al., 2023)
Tumor-suppressive role	Thyroid Cancer	TIAM1	-	Promote ferroptosis and inhibit progression	(Li W. et al., 2023)
Tumor-suppressive role	Gastric Cancer	PKMYT1	Promote mRNA stability	Inhibit proliferation, invasion, and migration	(Hu et al., 2022)
		WRAP53	Decrease mRNA stability and translation efficiency	Inhibit proliferation, invasion, and migration	(Zheng et al., 2025)
Tumor-suppressive role	Hepatocellular Carcinoma	PAQR4	Decrease mRNA and protein expression	Inhibit proliferation, invasion, and migration	(Wang W. et al., 2023)
Tumor-suppressive role	Pancreatic Cancer	PER1	Increase YTHDF2-dependent mRNA degradation	Inhibit proliferation, invasion, and migration	(Guo et al., 2020)
Tumor-suppressive role	Osteosarcoma	*Pre-miR-181b-1, YAP1*	Increase YTHDF2-dependent miRNA degradation and translation	Inhibit proliferation, migration, invasion, and survival	(Yu F. et al., 2021)
Tumor-suppressive role	Bladder Cancer	CSNK2A1	Enhance mRNA stability	Inhibit proliferation, invasion, and migration	(Yu H. et al., 2021)
Oncogene	Multiple Myeloma
		SAV1	Decrease mRNA stability and expression	Promote myeloma cell proliferation, neovascularization, invasion, and migration	(Yu et al., 2022)
Oncogene	Leukemia	ITPA	Enhance mRNA stability	Promote tumorigenesis and progression	(Li R. et al., 2023)
Oncogene	Acute Myeloid	*TACC3*	Enhance mRNA stability	Promote cancer cell proliferation, survival, and stem cell self-renewal	(Shen et al., 2020)
Oncogene	Renal Clear Cell Carcinoma	HNRNPDL	Promote nuclear translocation	Promote proliferation, migration, invasion, and cancer stemness	(Song et al., 2025)
Oncogene	Glioblastoma	SPP1	-	Promote cell proliferation and invasion	(Guan et al., 2023)
		NANOG	Enhance mRNA stability	Promote temozolomide resistance	(Guan et al., 2023)
		SOX2	Bind to SOX2 transcripts	Inhibit apoptosis, promote cell proliferation and temozolomide resistance	(Liu B. et al., 2020)
		CHK1, RAD51, FOXM1, YAP1	-	Promote radioresistance and invasiveness	(Kowalski-Chauvel et al., 2020)
		USP14	MST4-USP14-ALKBH5 signaling pathway	Promote radioresistance and tumorigenicity	(Zhou et al., 2025)
Oncogene	Melanoma	ABCA1	Decrease mRNA stability	Promote cell proliferation, colony formation, migration, and invasion, inhibit autophagy and promote tumor growth and metastasis	(Wang et al., 2024)
		MCT4	Enhance mRNA stability	Inhibit Treg and polymorphonuclear myeloidderived cells and promote tumor growth	(Li et al., 2020)
Oncogene	Glioma	G6PD	Enhance mRNA stability	Promote proliferation and energy metabolism	(Liu et al., 2021)
		ZDDHC3	Enhance mRNA stability	Promote PD-L1-mediated immune escape	(Tang et al., 2022)
Oncogene	Endometrial Cancer	IGF1R	-	Promote proliferation and invasion	(Pu et al., 2020)
Oncogene	Cervical Cancer	-	Reduce YTHDF2-dependent degradation	Promote tumor proliferation, migration, and invasion	(Liang et al., 2022)
Oncogene	Hepatocellular Carcinoma	MAP3K8	-	Promote proliferation, metastasis and PD-L1 macrophage recruitment	(You et al., 2022)
Oncogene	Colorectal Cancer	RAB5A	Reduce YTHDF2-dependent degradation	Promote tumor proliferation, migration, and invasion	(Shen et al., 2020)
		AXIN2	Dissociate from IGF2BP1	Drive immunosuppression and promote tumorigenesis	(Zhai et al., 2023)
Oncogene	Gastric	CHAC1	Enhance mRNA stability	Promote tumor growth and metastasis	(Chen Y. et al., 2023)
Oncogene	NSCLC	JAK2	Promote mRNA stability and translation	Induce PD-L1 expression, promote cell proliferation, migration and angiogenesis	(Hua et al., 2024)
		DDB2, XPC	Enhance mRNA stability	Reduce apoptosis and promote resistance to cisplatin	(Yu et al., 2025)
Oncogene	Head and Neck Squamous Cell Carcinoma	RIG-I	Reduce IFNα secretion	Promote immune escape and tumor progression	(Jin et al., 2022)
Oncogene	Intrahepatic Cholangiocarcinoma	PD-L1	Reduce YTHDF2-dependent degradation	Inhibit T cell expansion and cytotoxicity and promote immune escape	(Qiu et al., 2021)
Oncogene	Hepatocellular Carcinoma	TIRAP	-	Recruit monocytes and M2 polarization and inhibit radiosensitivity	(Chen C. et al., 2023)
Oncogene	Pancreatic Cancer	DDIT4-AS1	Enhance mRNA stability	Enhance cancer stemness and inhibit chemosensitivity to gemcitabine	(Zhang et al., 2022)
Oncogene	Triple Negative Breast Cancer	FOXO1	Enhance mRNA stability	Promote cancer stemness and adriamycin resistance	(Liu X. et al., 2024)
Oncogene	Epithelial Ovarian Cancer	JAK2	Reduce YTHDF2-dependent degradation	Promote tumor growth and cisplatin resistance	(Nie et al., 2021a)

A tumor-suppressive identity of ALKBH5 has been uncovered in select carcinoma types, wherein the enzyme exerts post-transcriptional control over transcripts encoding both cancer-promoting and growth-inhibitory factors. In non-small cell lung cancer (NSCLC), for instance, ALKBH5-driven destabilization of *CEMIP mRNA* curbs proliferative expansion while simultaneously priming tumor cells for enhanced susceptibility to paclitaxel-induced cell death (Gao et al., 2024). In pancreatic cancer, ALKBH5 promotes WIF-1 transcription and facilitates YTHDF2-dependent *PER1 mRNA* degradation, collectively suppressing proliferation, colony formation, and migration while improving gemcitabine chemosensitivity (Guo et al., 2020; Tang et al., 2020). The demethylase also attenuates hepatocellular carcinoma progression by reducing *PAQR4* mRNA and protein expression (Wang W. et al., 2023), and inhibits gastric cancer dissemination through destabilization of *PKMYT1* and *WRAP53* transcripts (Zheng et al., 2025). Furthermore, ALKBH5 confers radiosensitivity in colorectal cancer via *circAFF2* modulation (Shao et al., 2023), promotes ferroptosis in thyroid cancer through TIAM1 demethylation, and enhances TLR2 translation to suppress growth and induce apoptosis in hypopharyngeal squamous cell carcinoma (Ye et al., 2023). In osteosarcoma, ALKBH5 increases YTHDF2-dependent degradation of pre-miR-181b-1 and *YAP1* to inhibit survival and invasion (Yu F. et al., 2021; Yuan et al., 2021), whereas in bladder cancer, it enhances *CSNK2A1* mRNA stability to restrain malignant progression (Yu H. et al., 2021).

Conversely, ALKBH5 functions as an oncogenic driver across diverse hematologic and solid tumors by stabilizing pro-tumorigenic mRNAs and disrupting tumor-suppressive pathways. In acute myeloid leukemia and leukemia, ALKBH5 enhances *TACC3* (Shen et al., 2020) and *ITPA* (Liu et al., 2021) mRNA stability to promote cancer stem cell self-renewal and disease progression, while in multiple myeloma, it stabilizes TRAF1 to support cell survival (Qu et al., 2022) and destabilizes SAV1 to facilitate proliferation and neovascularization (Yu et al., 2022). In glioblastoma, ALKBH5 sustains stemness through NANOG and SRY-box transcription factor 2 (SOX2) (Guan et al., 2023; Liu B. et al., 2020), promotes cell proliferation and invasion via SPP1 (Guan et al., 2023), and drives radioresistance through upregulation of CHK1, RAD51, FOXM1, and YAP1 (Kowalski-Chauvel et al., 2020) as well as the USP14-ALKBH5 signaling axis (Zhou et al., 2025). Solid tumors including renal clear cell carcinoma (Song et al., 2025), melanoma (Li et al., 2020; Wang et al., 2024), glioma (Liu et al., 2021), endometrial cancer (Pu et al., 2020), and cervical cancer (Liang et al., 2022) harness ALKBH5 to enhance proliferation, immune evasion, and metastatic dissemination. In non-small cell lung cancer, ALKBH5 stabilizes Janus kinase 2 (JAK2) to induce PD-L1 expression and angiogenesis (Hua et al., 2024), while enhancing *DDB2* and *XPC* mRNA to confer cisplatin resistance (Yu et al., 2025). In the context of head and neck squamous cell carcinoma, ALKBH5 actively enhances tumor-mediated immunosuppressive programs, thereby fostering an environment conducive to immune escape (Jin et al., 2022) and intrahepatic cholangiocarcinoma (Qiu et al., 2021), drives macrophage recruitment and radioresistance in hepatocellular carcinoma (Chen C et al., 2023; You et al., 2022), and supports tumorigenesis and therapeutic resistance in colorectal cancer (Shen et al., 2020; Zhai et al., 2023), gastric cancer (Chen C et al., 2023), pancreatic cancer (Zhang et al., 2022), triple-negative breast cancer (Liu X. et al., 2024), and epithelial ovarian cancer (Nie et al., 2021a).

## 5 Small molecule targeted ALKBH5 therapy for cancer

### 5.1 ALKBH5 non-covalent inhibitor

#### 5.1.1 1-Aryl-1H-pyrazoles derivatives

Structure-activity relationship (SAR) of 1-aryl-1H-pyrazole derivatives against ALKBH5. As illustrated in Supplementary Table S1, systematic structural optimization of the hit compound **1** (IC_50_ = 4.253 μM), identified by fluorescence polarization (FP) assay, focused on the substituents of the 1-aryl ring (Region II, Figures 4a-c), affording a series of 1-aryl-1H-pyrazole-3-carboxylic acid derivatives (Figure 4d) (Fang et al., 2022). The FP assays were performed in triplicate using a FAM-labeled ssRNA substrate (5 nM) and recombinant human ALKBH5 (residues 66–292, 200 nM) in 50 mM HEPES buffer (pH 7.5) (Fang et al., 2022). Compared with compound **5**, modification at the para-position of the benzene ring with small hydrophobic groups improved the potency, as exemplified by 4-chloro (**10**, IC_50_ = 2.734 μM) and 4-bromo (**7**, IC_50_ = 0.591 μM) (Fang et al., 2022). By contrast, large hydrophobic (4-isopropyl, **15**, IC_50_ = 6.615 μM). Notably, relocation of an electron-withdrawing group to the meta-position markedly enhanced the inhibitory activity: the 3-bromo analogue **8** achieved an IC_50_ of 0.727 μM, whereas the 3-trifluoromethyl derivative **3** emerged as the most potent inhibitor in this series (IC_50_ = 0.021 μM). The 3-methyl analogue **12** displayed only modest activity (IC_50_ = 4.944 μM) (Fang et al., 2022). Disubstitution at both the meta- and para-positions further refined the SAR: 4-bromo-3-trifluoromethyl (**5**, IC_50_ = 0.421 μM), 3-bromo-4-methyl (**6**, IC_50_ = 0.559 μM), 4-bromo-3-methyl (**2**, IC_50_ = 0.061 μM), and 4-methyl-3-trifluoromethyl (**4**, IC_50_ = 0.396 μM) all exhibited substantially improved potency compared with their monosubstituted counterparts, underscoring that simultaneous occupancy of the meta-position hydrophobic sub-pocket and the para-position periphery is highly favorable (Fang et al., 2022; Wang, Y. et al., 2023).

**FIGURE 4:**
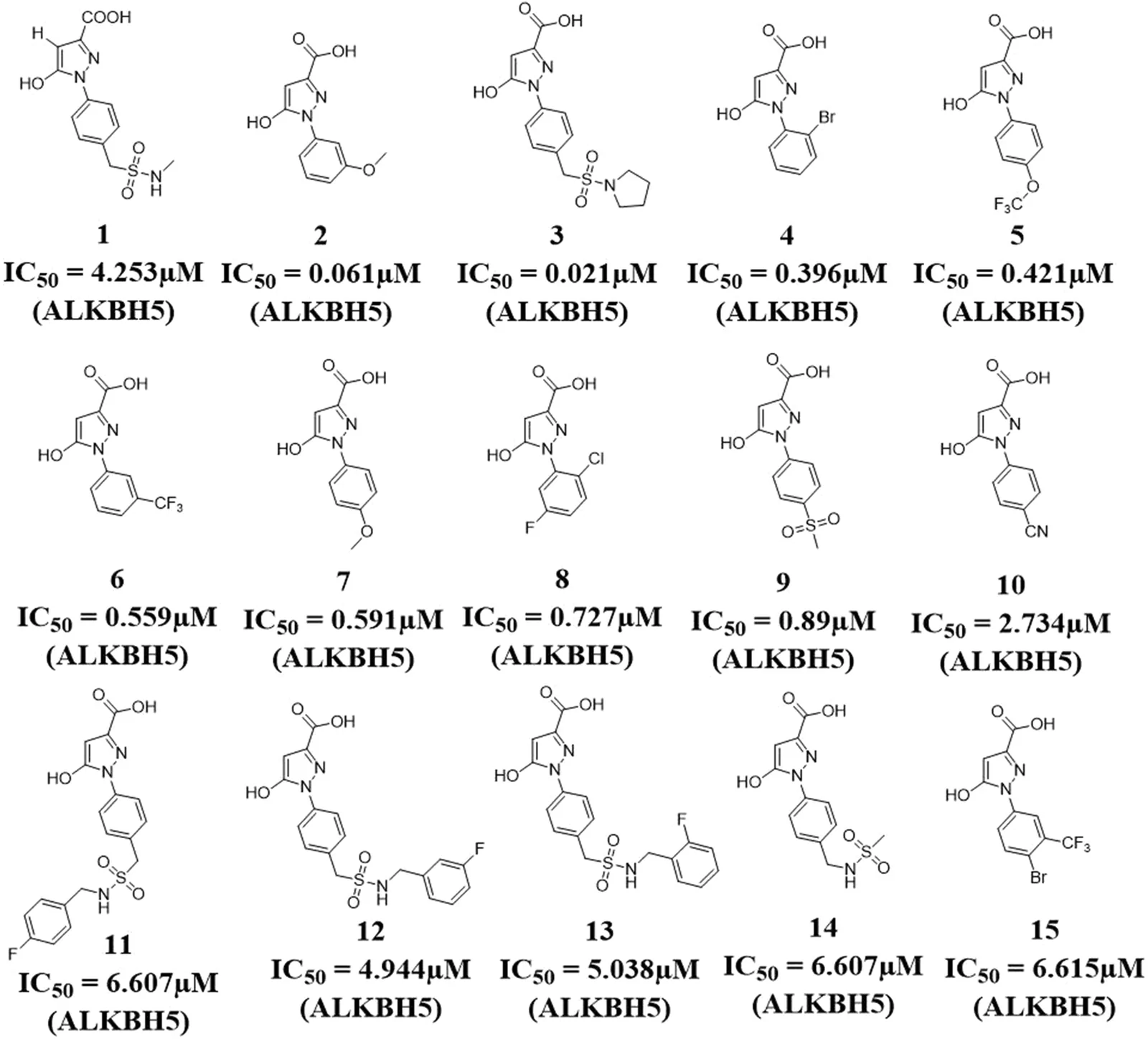
The chemical structure and corresponding ALKBH5 biological activity of 1-Aryl-1H-pyrazoles derivatives compound 1–15.

As depicted in Figure 5, molecular docking analysis revealed that all fifteen 1-aryl-1H-pyrazole derivatives (compounds **1–15**) adopted a conserved binding orientation within the active pocket of ALKBH5, yet established distinct hydrogen-bonding networks with surrounding residues that rationalize their differential inhibitory potencies. Compound **1** (Figure 5a) simultaneously formed hydrogen bonds with Asp206, Ser217, Lys132, and Ser219, establishing a foundational interaction pattern for this series. Compounds **2–4** (Figures 5b-d) maintained critical polar contacts with Arg277, Asp206, His266, Lys132, and Asn193, thereby preserving the core interactions. Compounds **5–8** (Figures 5e-h) continued to engage Arg277, Asn193, Asp206, and Lys132, with compound **6** additionally interacting with Ser217. Notably, compound **9** (Figure 5i) displayed the most extensive hydrogen-bonding network among the series, forming multiple polar interactions with Lys132, Asn193, Ser219, Ser217, and Arg277, which likely contributes to its enhanced binding affinity. Compounds **10** and **11** (Figures 5j,k) established hydrogen bonds with Asn193, Arg277, His266, and Lys132. Compounds **12–15** (Figures 5l-o) gained additional stabilization through interactions with Arg277, Lys132, His266, His204, Tyr139, Arg283, and Ser217/Ser219. Collectively, these docking results demonstrate that while Arg277, Asp206, and Lys132 serve as conserved anchor residues for the entire series, the variable hydrogen-bonding engagements with Asn193, His266, Ser217, Ser219, Tyr139, His204, and Arg283 constitute the primary structural determinants modulating the binding strength and inhibitory activity of these ALKBH5 non-covalent inhibitors.

**FIGURE 5:**
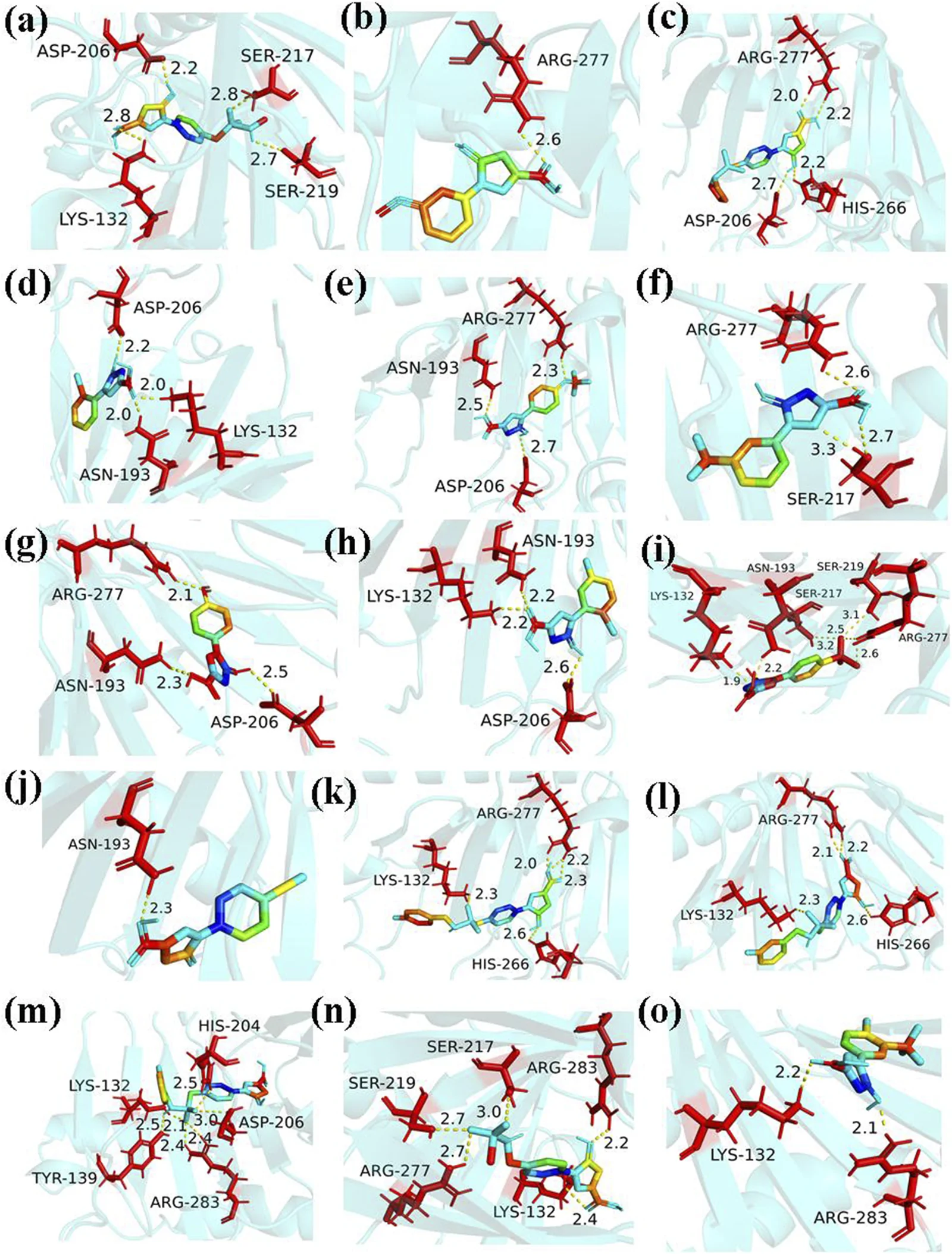
Predicted binding modes of 1-aryl-1H-pyrazole derivatives 1–15 within the ALKBH5 active pocket (PDB: 4NRO) derived from molecular docking simulations. **(a)** 1, **(b)** 2, **(c)** 3, **(d)** 4, **(e)** 5, **(f)** 6, **(g)** 7, **(h)** 8, **(i)** 9, **(j)** 10, **(k)** 11, **(l)** 12, **(m)** 13, **(n)** 14, **(o)** 15. Amino acid residues critical for ligand recognition are shown as red sticks; putative hydrogen bonds are denoted by yellow dashed lines with distances in angstroms (Å).

Per Fang et al., a comprehensive series of biochemical and cellular assays was executed to validate the pharmacological properties of compound 3 (Supplementary Table S2) (5-hydroxy-1-(3-(trifluoromethyl)phenyl)-1H-pyrazole-3-carboxylic acid), the optimized lead candidate (Fang et al., 2022). Biophysically, compound **3** displayed potent inhibition against ALKBH5 in a fluorescence polarization (FP) assay with an IC_50_ value of 0.021 ± 0.009 μM, and differential scanning fluorimetry (DSF) confirmed dose-dependent thermal stabilization of the target protein (ΔT_m_ = 8.75 °C) (Fang et al., 2022). Mechanistically, an *in vitro* dot blot demethylation assay demonstrated that compound **3** efficiently blocked ALKBH5-mediated removal of m^6^A marks in a concentration-dependent manner. The compound also exhibited high selectivity, showing negligible activity against the paralogue FTO and other AlkB subfamily members (ALKBH1, ALKBH2) in both DSF and FP formats (Fang et al., 2022). Of particular note regarding cellular pharmacology, compound **3** possesses favorable membrane permeability and robust intracellular target engagement, as evidenced by a cellular thermal shift assay (CETSA) showing significant stabilization of ALKBH5 in HepG2 cells compared to DMSO-treated controls (Fang et al., 2022). Furthermore, dot blot analysis of total RNA isolated from HepG2 cells treated with compound **3** revealed a marked, dose-dependent increase in global m^6^A abundance, confirming that the inhibitor effectively suppresses ALKBH5 demethylase activity in a physiologically relevant intracellular environment. Taken together, these data establish compound **3** as a potent, highly selective, and cell-permeable ALKBH5 inhibitor capable of elevating m^6^A levels in intact cells, supporting its utility as a chemical probe for exploring the biological functions of ALKBH5 (Fang et al., 2022).

#### 5.1.2 Pyrazolo [1,5-a]pyrimidine derivatives

As shown in Figure 6, a series of pyrazolo [1,5-a]pyrimidine derivatives (16–21) were designed and synthesized to investigate the structure–activity relationship (SAR) against ALKBH5 (Wang Y. et al., 2023). All compounds share a common pyrazolo [1,5-a]pyrimidine core scaffold, with variations at the C-1 position side chain and the phenyl ring substitution (R^1^ = H or OH) (Wang Y. et al., 2023). The preliminary SAR analysis revealed that the introduction of a hydroxyl group at the R^1^ position consistently improved the inhibitory activity across all side-chain types: in Supplementary Table S1, compounds **17** (R^1^ = -OH, IC_50_ = 14.44 μM), **19** (IC_50_ = 7.88 μM), and **21** (IC_50_ = 2.97 μM) exhibited 1.3-, 1.2-, and 1.6-fold enhanced potency relative to their R^1^ = H counterparts **16** (IC_50_ = 18.59 μM), **18** (IC_50_ = 9.12 μM), and **20** (IC_50_ = 4.64 μM), respectively (Wang Y. et al., 2023). More significantly, optimization of the C-3 side chain markedly influenced the activity profile. Replacement of the chlorophenyl group (**16/17**) with ester functionalities (**18/19**) reduced the IC_50_ values from double-digit to single-digit micromolar concentrations. Further extension of the ester side chain to incorporate a CF_3_-containing biphenyl motif (**20/21**) substantially boosted the ALKBH5 inhibitory potency. Optimization efforts ultimately yielded compound **21** as the most powerful inhibitor within the chemical series. The compound registered an IC_50_ of 2.97 μM, reflecting a 6.3-fold leap in activity compared with the initial lead compound **16**. These results demonstrate that the synergistic modification of the phenyl ring hydroxyl group and the CF_3_-biphenyl ester side chain at the C-3 position of the pyrazolo [1,5-a]pyrimidine scaffold is a critical strategy for enhancing ALKBH5 inhibitory activity (Wang Y. et al., 2023).

**FIGURE 6:**
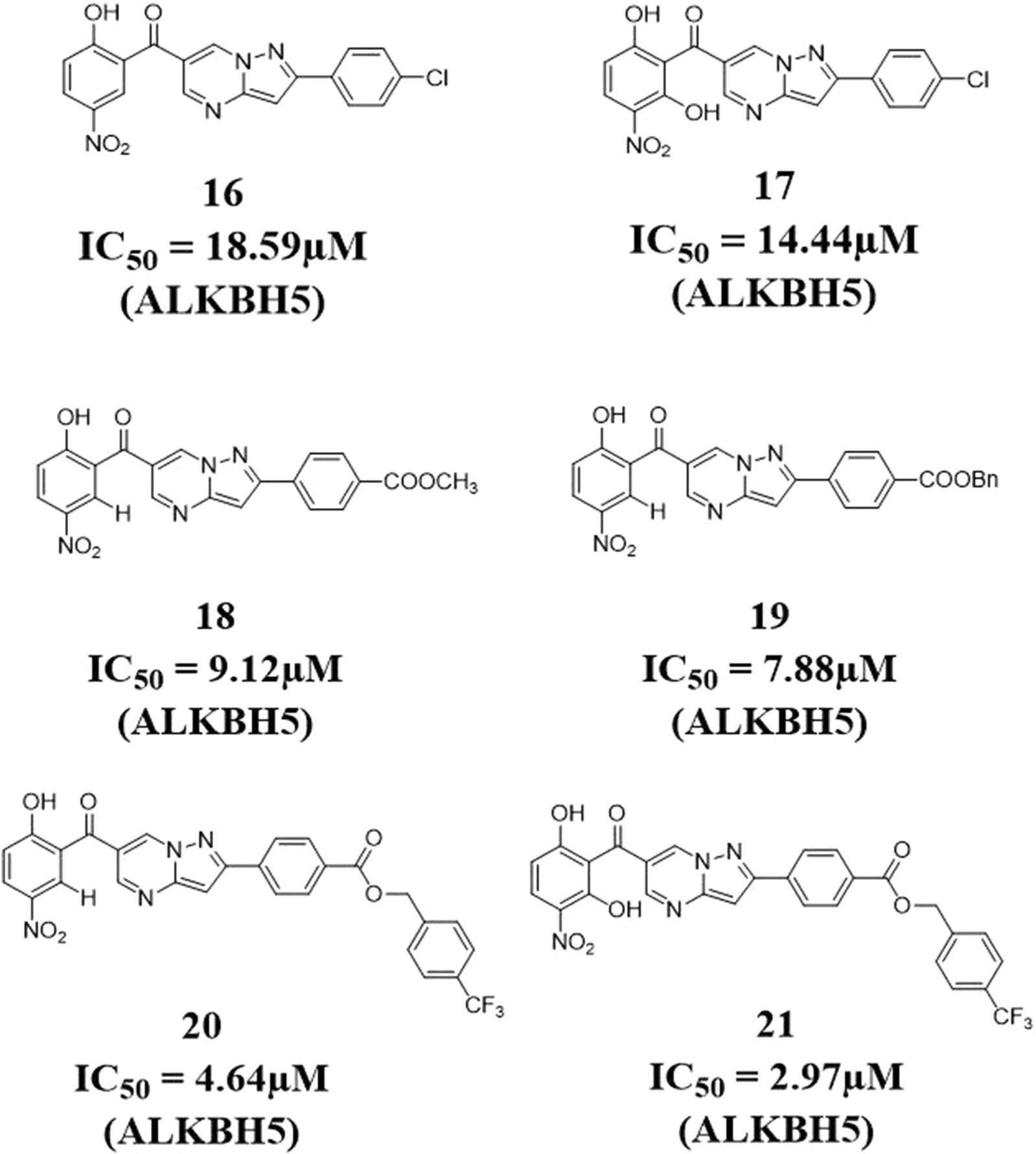
Chemical structures and ALKBH5 inhibitory activities of pyrazolo [1,5-a]pyrimidine derivatives 16–21.

To elucidate the binding mode of pyrazolo [1,5-a]pyrimidine derivatives with ALKBH5 at the molecular level, molecular docking studies were performed using the ALKBH5 crystal structure. As depicted in Figure 7, compounds **16–21** all bind within the 2-oxoglutarate (2-OG) active site pocket of ALKBH5, yet exhibit distinct interaction patterns dictated by their side-chain architectures. Compound **16** (Figure 7a) establishes key hydrogen bonds with ARG-130 (2.6 Å) and ASP-206 (3.1 Å) through its pyrazolo [1,5-a]pyrimidine core and chlorophenyl moiety. The introduction of the phenolic hydroxyl group in **17** (Figure 7b) enables an additional hydrogen bond interaction with VAL-202 (2.2 Å) while maintaining contacts with ASP-206 (3.2 Å) and ARG-130 (2.8 Å, 2.6 Å). Compounds **18** (Figure 7c) and **19** (Figure 7e), bearing ester side chains, form extended hydrogen-bonding networks with ASP-206, VAL-202, ARG-130, and ARG-277, which correlates with their improved inhibitory potencies. Remarkably, compounds **20** (Figure 7d) and **21** (Figure 7f), equipped with the CF_3_-biphenyl ester side chains, penetrate deeper into the ALKBH5 binding pocket and engage in multiple favorable interactions with pocket residues including ARG-126, VAL-124, GLU-121, HIS-122, TYR-139, and ASP-125. Specifically, **20** forms an extensive hydrogen-bond network with HIS-122 (1.9 Å, 2.6 Å), GLU-121 (2.2 Å), and ASP-125 (3.3–3.5 Å), while **21** forms a strong hydrogen bond with ASP-125 (2.4 Å) alongside hydrophobic contacts with the CF_3_-biphenyl tail. These docking results rationalize the observed SAR trends: the progressive extension and optimization of the C-1 side chain allow the pyrazolo [1,5-a]pyrimidine derivatives to occupy additional subsites within the ALKBH5 active pocket, thereby establishing more robust intermolecular interactions and significantly enhancing inhibitory efficacy.

**FIGURE 7:**
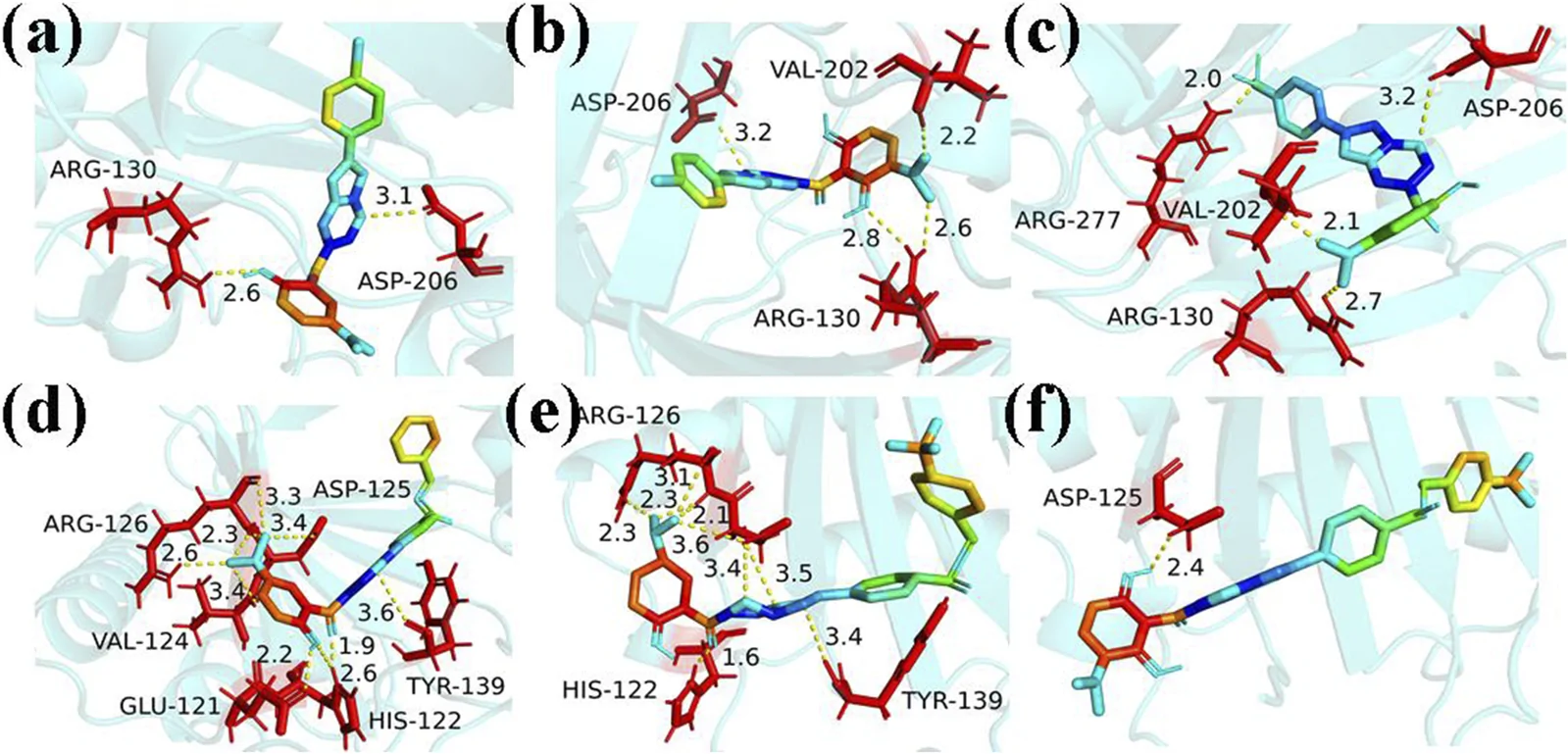
The computationally predicted binding geometries of pyrazolo[1,5-a]pyrimidine inhibitors 16-21 complexed with the ALKBH5 (PDB Code: 4NRO) active site are illustrated in panels **(a)** through **(f)**: compound 16 **(a)**, 17 **(b)**, 18 **(c)**, 20 **(d)**, 19 **(e)**, and 21 **(f)**. Hydrogen bonding networks are visualized as yellow dashed traces, with interatomic distances noted in angstroms (Å). Residues of structural significance for ligand-protein engagement are explicitly tagged.

In cellular assays, compound 21 (Supplementary Table S2) elevated global m^6^A levels in AML cells, suppressed the proliferation of MOLM-13 and MV4-11 cell lines, achieving IC_50_ values of 0.45 μM and 1.2 μM, respectively, and showed low toxicity toward normal HEK293 cells and human umbilical vein endothelial cells (HUVECs). Furthermore, compound **21** induced G0/G1 cell cycle arrest and concentration-dependent apoptosis, mechanistically acting through the ALKBH5-TACC3-c-Myc signaling axis by reducing TACC3 mRNA stability and downstream protein expression. These results collectively establish compound **21** as a potent, selective, and cell-active ALKBH5 inhibitor with a distinct non-2-OG competitive mechanism.

#### 5.1.3 Pyrazolone and benzodioxole derivatives

An extensive high-throughput screening campaign was conducted utilizing the Enamine library of pharmacologically annotated small molecules, two novel hit compounds, **Ena15** and **Ena21** (Supplementary Table S1; Supplementary Table S2), were identified as selective inhibitors of ALKBH5 (Takahashi et al., 2022). As summarized in Figure 8a, **Ena15** inhibited ALKBH5 with an IC_50_ value of 18.3 ± 1.8 μM, whereas **Ena21** displayed comparable potency with an IC_50_ of 15.7 ± 1.0 μM (Takahashi et al., 2022). Notably, both compounds exhibited divergent selectivity profiles against the homologous demethylase FTO (Takahashi et al., 2022). At 80 μM, **Ena21** showed negligible inhibitory activity toward FTO (36% inhibition), whereas **Ena15** not only failed to inhibit FTO but instead enhanced its demethylase activity (−130% inhibition), indicating that both molecules are selective ALKBH5 inhibitors with distinct off-target behaviours (Takahashi et al., 2022).

**FIGURE 8:**
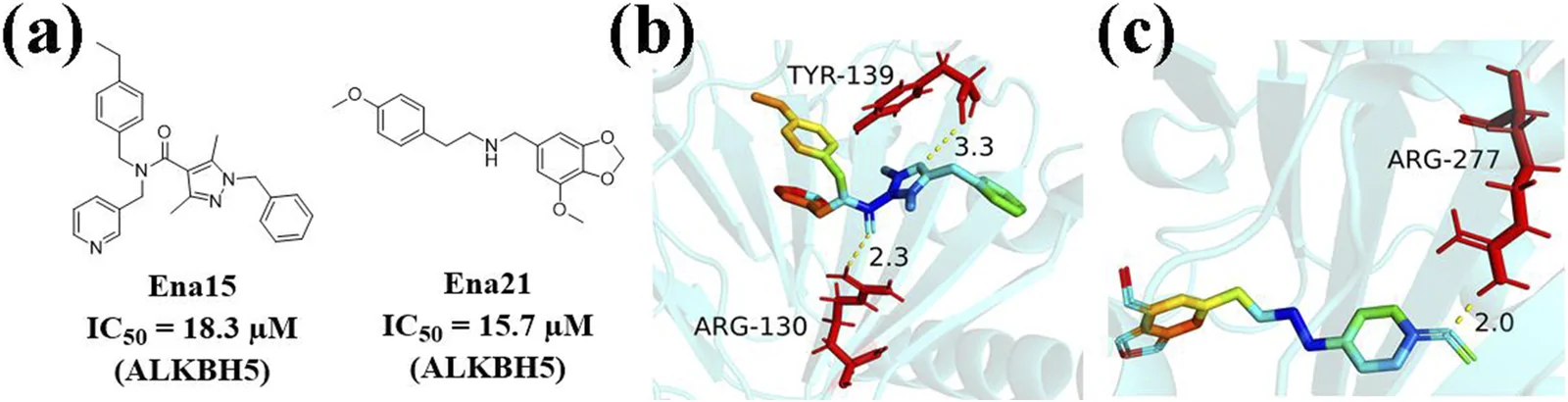
Chemical structures and predicted binding modes of pyrazolone and benzodioxole derivatives. **(a)** Structures of Eual5 and Eua21 against ALKBH5. **(b)** Docking pose of Eual5 within the ALKBH5 active site (PDB: 4NRO). **(c)** Docking pose of Eua21 within the ALKBH5 active site (PDB: 4NRO). Yellow dashed lines denote hydrogen bonds with distances given in angstroms (Å).

Molecular docking simulations were subsequently performed to rationalize the binding behaviors of **Eual5** and **Eua21** within the ALKBH5 active pocket. As depicted in Figure 8b, the pyrazolone derivative **Eual5** anchors in the active site through a network of hydrogen-bonding interactions, specifically engaging ARG-130 (2.3 Å) and TYR-139 (3.3 Å) via its pyrazolone carbonyl and neighboring heteroatoms. Conversely, the benzodioxole derivative **Eua21** adopts an extended conformation that projects its aromatic tail toward a distinct subsite, establishing a hydrogen bond with ARG-277 (2.0 Å) through the terminal methoxyphenyl or benzodioxole oxygen atoms (Figure 8c). The divergent interaction profiles of the two chemotypes, **Eual5** relying on contacts with the central residues and **Eua21** exploiting a peripheral arginine anchor, mirror their comparable yet modest inhibitory potencies. Collectively, these docking analyses reveal that although both scaffolds successfully occupy the ALKBH5 active pocket, the limited number of specific polar interactions and the absence of deep hydrophobic burial indicate substantial room for structure-guided optimization to strengthen ligand-protein affinity.

#### 5.1.4 Imidazobenzoxazine-5-thione derivatives

Starting from the lead compound MV1035, a tricyclic imidazobenzoxazine-5-thione previously shown to inhibit both ALKBH2 and ALKBH5, a series of analogues were designed to dissect the key structural motifs governing activity (Figure 9) (Rivara et al., 2024). These findings highlight the ortho-phenol and the propyl/methyl imidazole substituents as indispensable pharmacophoric elements, with compound **22** [2-(4-methyl-5-propyl-1H-imidazole-2-yl)phenol] (Supplementary Table S1)emerging as the most potent analogue in the panel (Rivara et al., 2024).

**FIGURE 9:**
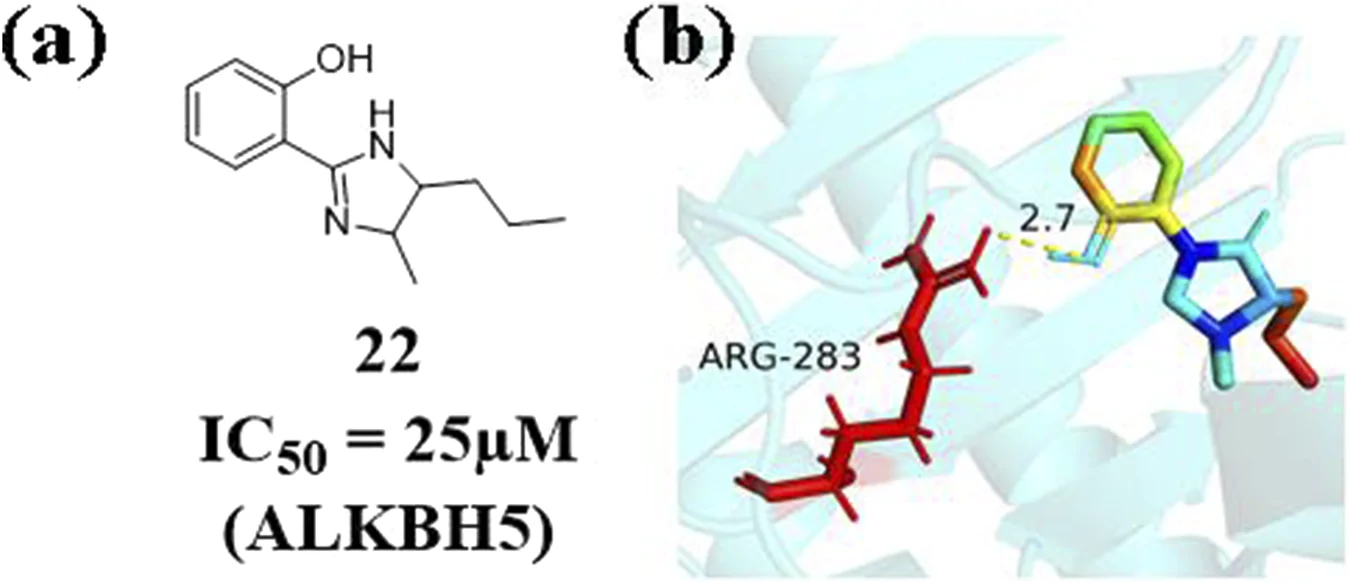
**(a)** Structures of 22 against ALKBH5. **(b)** Docking pose of 22 within the ALKBH5 active site (PDB: 4NRO).

The interaction landscape of imidazobenzoxazine-5-thione derivatives with ALKBH5 was explored through docking calculations. As shown in Figures 9a,b, the inhibitor adopts a pose in which its thione unit lies within hydrogen-bonding range (2.7 Å) of Arg283, a residue whose guanidinium side chain furnishes a critical electrostatic tether. This polar linkage, together with the snug insertion of the fused benzoxazine–imidazole bicycle into the catalytic cleft, underlies the favorable binding energetics. Surrounding residues contribute additional hydrophobic/van der Waals forces that cradle the planar aromatic surface of the ligand. Moreover, the docking model suggests that substituent variations on the heterocyclic platform alter the degree of shape complementarity toward a nearby hydrophobic microenvironment, thereby modulating inhibitory potency. Such computational evidence aligns with the empirical SAR, pointing to a dual requirement—precise H-bonding to Arg283 and extensive nonpolar burial of the fused ring—for achieving high-affinity ALKBH5 blockade.

Guided by the previously reported putative binding mode of MV1035 with ALKBH5, which partially overlaps the 2-oxoglutarate pocket compound **22** was selected for comprehensive biochemical and cellular validation (Rivara et al., 2024). In cell-free demethylase assays, **22** (Supplementary Table S2) dose-dependently inhibited ALKBH2 with an approximate IC_50_ of 25 μM and markedly suppressed ALKBH5 activity, evidenced by a strong, concentration-dependent increase in m^6^A-RNA signal in dot-blot analyses (10–50 μM). In U87-MG glioblastoma cells, **22** significantly reduced viability in a time- and dose-dependent manner, outperforming the parent MV1035 at 50 μM after 72 h (Rivara et al., 2024). These results establish **22** as a simplified yet effective phenylimidazole lead that retains dual ALKBH2/ALKBH5 inhibitory activity, offering a new starting point for the development of glioblastoma-targeted epitranscriptomic agents (Rivara et al., 2024).

#### 5.1.5 5-Carboxy-8-hydroxyquinoline derivatives

ALKBH5, an α-KG-dependent demethylase that removes m6A modifications from RNA as part of the “eraser” machinery, is pharmacologically blocked by the small synthetic compound **IOX1** (Figure 10). By selectively blocking the enzymatic activity of ALKBH5, **IOX1** restores global m^6^A methylation levels and subsequently modulates downstream metabolic reprogramming (Cheng et al., 2022). In the context of acute myocardial infarction (AMI), **IOX1** was identified as a potential therapeutic agent to counteract ALKBH5-overexpression-induced suppression of tricarboxylic acid (TCA) cycle dehydrogenases and dysregulation of associated amino acid substrates (Cheng et al., 2022). To enable targeted delivery to the infarct region, **IOX1** was encapsulated within a surface-modified bioengineered ferritin nanocage (HSSS, HAfFtO-ST-SC-Sf) to generate the nanocomplex HSSS-I, though free **IOX1** was also evaluated independently for its pharmacological efficacy (Cheng et al., 2022).

**FIGURE 10:**
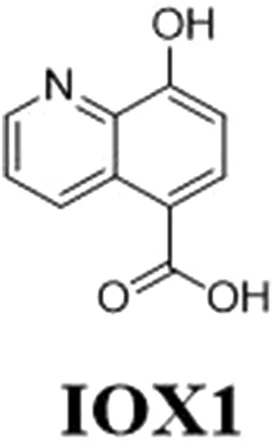
The structure of IOX1.

The ALKBH5 inhibitory activity of **IOX1** (Supplementary Table S2) was investigated through *in vitro* cellular assays and *in vivo* animal studies (Cheng et al., 2022). To model I/R pathology, H9C2 cardiomyocytes and rat aortic endothelial cells (RAOEC) were exposed to H2O2 or subjected to hypoxia followed by reoxygenation. Under these conditions, **IOX1** treatment effectively counteracted the decline in m6A RNA methylation markers induced by simulated ischemia/reperfusion and normalized the operational capacity of essential TCA cycle enzymes such as fumarase, isocitrate dehydrogenase, and malate dehydrogenase, and normalized the altered concentrations of related amino acid metabolites (Cheng et al., 2022). Furthermore, **IOX1** markedly improved cell viability, attenuated apoptosis, and reduced the cell death ratio (Cheng et al., 2022). In a Sprague-Dawley rat AMI model established by left anterior descending coronary artery ligation and reperfusion, intravenous administration of **IOX1** (10 mg/kg, caudal vein, once daily for 14 days) substantially decreased serum levels of the myocardial injury markers creatine kinase-MB (CK-MB) and lactate dehydrogenase (LDH), cardiac contractile and diastolic performance were significantly rescued following **IOX1** treatment, manifesting as improved left ventricular ejection fraction (LVEF) and left ventricular fractional shortening (LVFS) measurements and a favorable shift in the E/A waveform ratio. The hemodynamic profile was concurrently stabilized, featuring optimized LVSP, reduced ventricular filling pressure (LVEDP), and superior pressure derivative kinetics (±dP/dt). Tissue-level protection was further demonstrated by contracted infarct dimensions, attenuated fibrotic remodeling, and diminished apoptotic burden, as independently verified through Evans blue-TTC viability staining, Masson’s trichrome connective tissue staining, and TUNEL apoptotic labeling (Cheng et al., 2022).

### 5.2 ALKBH5 covalent inhibitor

#### 5.2.1 Salicylaldehyde derivatives

Starting from the reference inhibitor **DDO-2728** (Figure 11; Supplementary Table S1, IC_50_ = 4.06 μM), a scaffold-hopping strategy was pursued in which the triazole-trifluoromethylphenyl fragment was replaced by a picolinic acid-sulfonamide motif, affording compound **23** (Fei et al., 2025). This structural truncation, however, proved detrimental to potency (IC_50_ = 7.44 μM), indicating that the abbreviated framework alone could not sustain robust target engagement (Fei et al., 2025). Subsequent installation of an ortho-hydroxy and formyl group on the phenyl ring of **23** to generate the salicylaldehyde pharmacophore in compound **24** restored inhibitory efficacy (IC_50_ = 3.40 μM), representing an approximately 2.2-fold gain over **23** and underscoring the critical role of the salicylaldehyde signature in affinity enhancement (Fei et al., 2025). Expanding the aromatic surface from a phenyl to a naphthalene core in compound **25** furnished the most potent analogue within this chemotype (IC_50_ = 1.43 μM), improving activity by roughly 2.8-fold relative to the parental **DDO-2728** (Fei et al., 2025). Conversely, merely repositioning the hydroxy and formyl substituents around the naphthalene scaffold in regioisomer **26** triggered a severe collapse in activity (IC_50_ = 8.56 μM), despite retention of identical functional elements. Collectively, these data reveal that the geometric deployment of the salicylaldehyde motif, rather than its mere presence, governs high-affinity ALKBH5 blockade (Fei et al., 2025).

**FIGURE 11:**
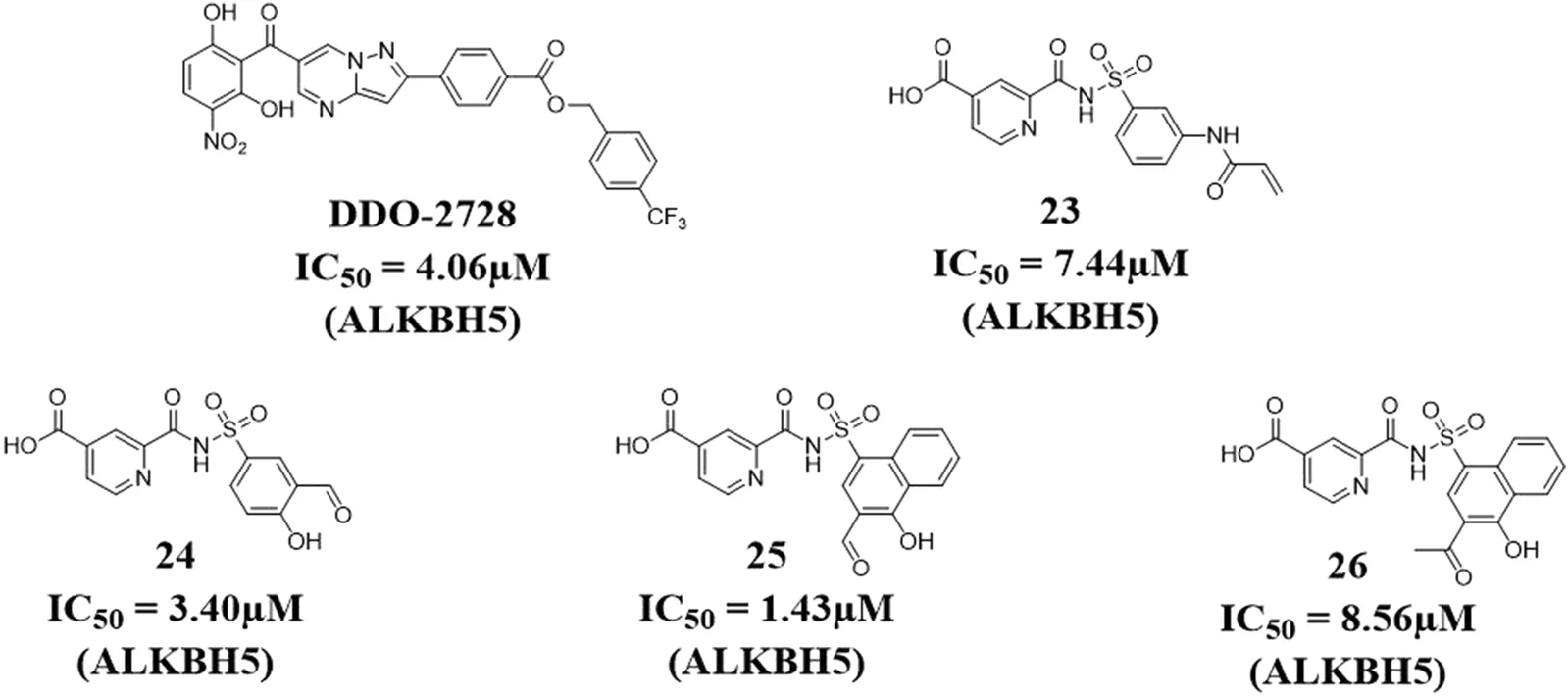
Design and chemical structures of salicylaldehyde derivatives 23–26 and the reference inhibitor DDO-2728, together with their inhibitory activities against ALKBH5 as determined by fluorescence polarization (FP) assay. The IC_50_ values represent the mean of at least three independent experiments.

To dissect the atomic underpinnings of inhibition, docking simulations were conducted with representative members of the series. As illustrated in Figure 12, compound **25** settled into a favorable pose within the ALKBH5 active pocket: the phenolic -OH engaged in hydrogen bonding with TYR-139 (2.2 Å) and ASN-193 (1.9 Å), whereas the aldehyde oxygen simultaneously anchored to the histidine dyad HIS-204 (2.2 Å) and HIS-266 (2.5 Å). The naphthalene nucleus, in turn, nestled against TYR-195 (2.5 Å) and ARG-283 (2.4 Å and 3.1 Å), exploiting both π–π stacking and extensive van der Waals contacts that collectively rationalize its superior potency. By contrast, the altered spatial arrangement in compound **26** disrupted this interaction network, it could only sustain tenuous hydrogen-bond contacts with LYS-132 (2.6 Å), TYR-139 (2.0 Å), and ARG-283 (2.3 Å and 2.5 Å), while forfeiting the crucial histidine-mediated anchoring entirely, an observation that accounts for its markedly attenuated binding. Compound **24**, carrying the phenyl-based salicylaldehyde warhead, retained hydrogen-bonding capacity toward ASN-193 (2.0 Å), ARG-277 (2.2 Å), and ASP-206 (2.4 Å), yet lacked the extended conjugation and hydrophobic bulk of the naphthalene variant, resulting in intermediate activity bracketed between **25** and **26**. Taken together, the computational evidence points to a dual recognition mechanism: hydrogen-bond tethering of the salicylaldehyde group to the catalytic histidine pair (HIS-204 and HIS-266), coupled with deep hydrophobic accommodation of the naphthalene ring, serves as the structural cornerstone for potent ALKBH5 inhibition within this derivative class.

**FIGURE 12:**
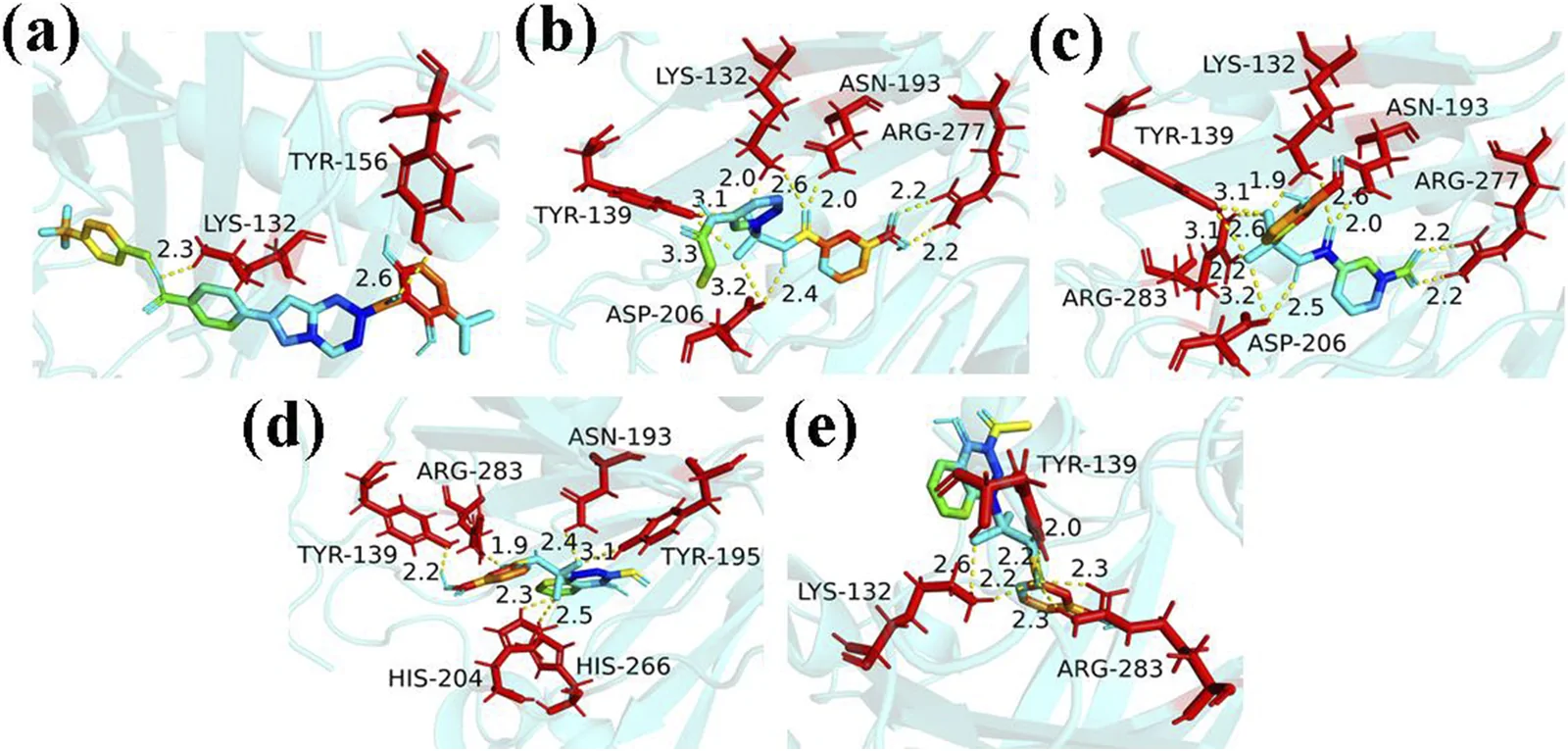
Predicted binding modes of DDO-2728 **(a)**, compound 23 **(b)**, compound 24 **(c)**, compound 25 **(d)**, and compound 26 **(e)** within the active pocket of ALKBH5(PDB: 4NRO) derived from molecular docking simulations. Amino acid residues critical for ligand recognition are displayed as red sticks, and putative hydrogen-bonding interactions are denoted by yellow dashed lines with interatomic distances annotated in angstroms (Å).

The optimized covalent inhibitor 25 (Supplementary Table S2) was extensively characterized by molecular docking and cellular assays (Fei et al., 2025). Covalent docking against ALKBH5 revealed that the aldehyde group of **25** forms an imine bond with Lys132. Isothermal titration calorimetry (ITC) confirmed direct binding with a K_d_ of 2.80 ± 0.55 μM (Fei et al., 2025). Drug affinity responsive target stability (DARTS) and cellular thermal shift assays (CETSA) in MOLM-13 cells further validated target engagement in both cell lysates and living cells (Fei et al., 2025). Mass spectrometry and HPLC-MS/MS unequivocally demonstrated covalent modification of Lys132, while the K132E mutant showed markedly reduced binding and inhibition, corroborating Lys132 as the specific covalent target. **25** exhibited excellent selectivity for ALKBH5 (FP IC_50_ = 0.49 ± 0.05 μM) over FTO (IC_50_ = 14.16 ± 1.68 μM) and ALKBH3 (IC_50_ = 11.71 ± 0.12 μM), and its inhibitory activity was independent of 2-OG concentration (Fei et al., 2025). Functionally, **25** elevated cellular m^6^A levels in MV4-11 cells, downregulated the ALKBH5-AXL signalling axis (reducing AXL mRNA and protein abundance), promoted G1-stage growth cessation, preventing further cell division, and promoted apoptosis in MOLM-13 cells, underscoring its potential as a selective chemical probe for ALKBH5 (Fei et al., 2025).

#### 5.2.2 Oxindole derivatives

Encouraged by the promising inhibitory activity of the virtual screening hit AG-690/34437009, a focused library of oxindole derivatives (Figure 13; Supplementary Table S1; Supplementary Tables S2, S27-S32) was rationally designed and synthesized to systematically probe the structure-activity relationships (SAR) and enhance ALKBH5 inhibitory potency (Yang et al., 2025). Removal of the phenolic hydroxyl group afforded **27** (IC_50_ = 5.027 μM), which exhibited enhanced potency relative to the parent compound. SAR studies revealed that the 3-bromo-5-nitrophenyl motif is indispensable for activity: Relocating the bromo substituent from the 3- to the 4-position (**28**, IC_50_ = 8.532 μM) diminished activity, indicating that the 3-bromo group is optimal for engaging L129 via a second hydrogen bond. Replacement of the 4-chlorophenyl ring with a methoxyl group at the 3- or 4-position yielded **29** (IC_50_ = 5.608 μM) and **30** (IC_50_ = 3.848 μM), respectively (Yang et al., 2025). Finally, substitution of the nitro and bromo groups with cyano and fluoro atoms (**31** and **32**) resulted in reduced activity (IC_50_ = 6.126 and 8.663 μM, respectively), underscoring the superiority of the 3-bromo-5-nitro combination. Collectively, **30** emerged as the most potent inhibitor within this series (Yang et al., 2025).

**FIGURE 13:**
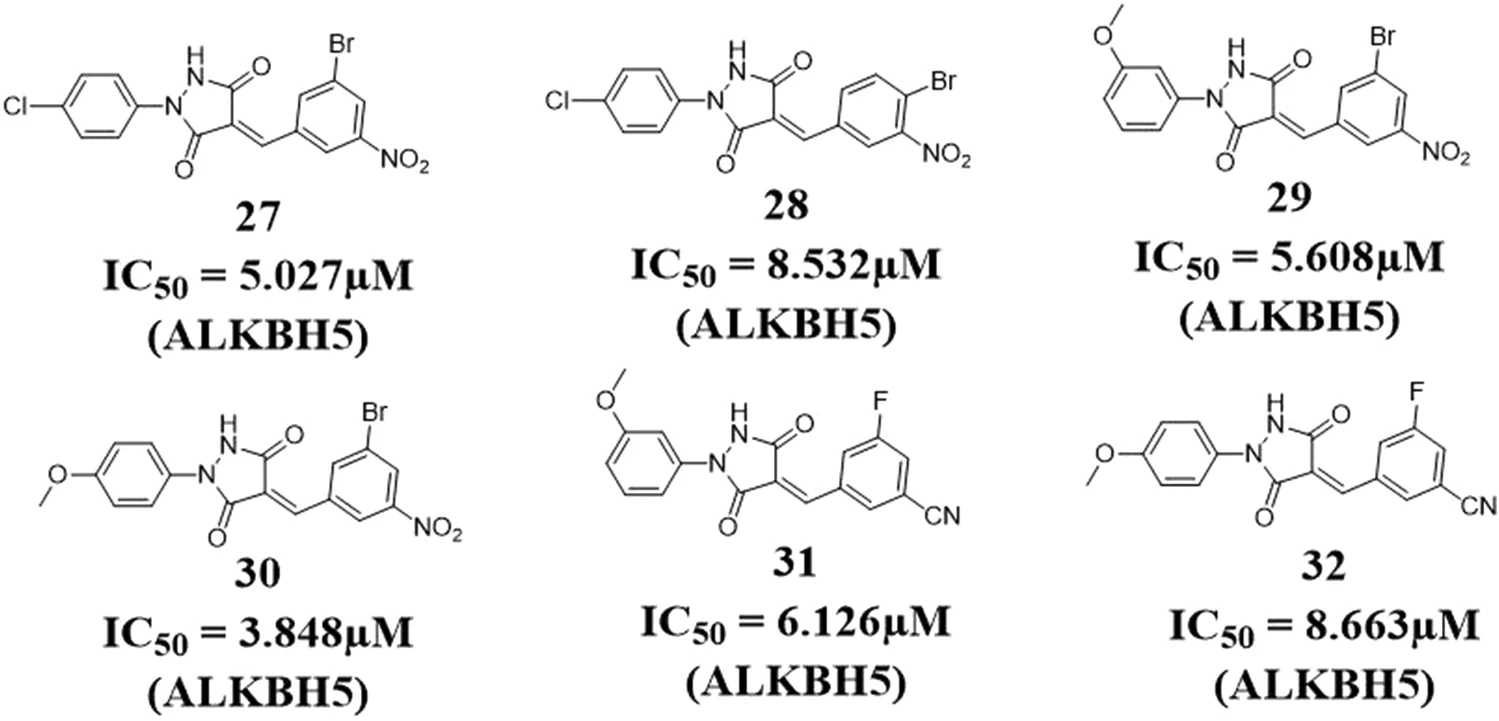
Chemical structures and ALKBH5 inhibitory activities of the designed oxindole derivatives. ALKBH5 inhibitory activities of target compounds 27–32 determined by the fluorescence polarization (FP) assay.

To rationalize the observed structure-activity relationships at the molecular level, To gain insight into how oxindole-based compounds engage the ALKBH5 binding cavity, molecular docking analyses were carried out to map their preferred poses and interaction geometries. As depicted in Figure 14, the oxindole scaffold is consistently anchored in the metal-binding site through a conserved hydrogen-bonding network that mimics the endogenous α-ketoglutarate cofactor. The C2 carbonyl oxygen of the oxindole lactam acts as a hydrogen-bond acceptor to the side-chain amide of Asn193 (2.0–2.6 Å) and/or the carboxylate of Asp206 (2.3–3.6 Å), while the indole NH donates a hydrogen bond to Asp206 or Arg283 (2.2–2.8 Å), thereby locking the inhibitor in a productive orientation. The 3-aryl substituent extends into a hydrophobic sub-pocket lined by Tyr139, His266, and Arg283, where π-π stacking and van der Waals contacts provide substantial binding energy. Notably, the more potent analogues bear additional polar functionalities on the terminal phenyl ring that engage in auxiliary hydrogen-bonding interactions with Ser217, Ser219, Thr254, or Glu153 (2.1–3.1 Å), effectively bridging the ligand to the solvent-exposed loop region and enhancing affinity. Conversely, analogues lacking these distal polar groups or carrying sterically demanding substituents fail to establish these supplementary contacts, resulting in weaker docking scores and reduced enzymatic inhibition, which is consistent with their attenuated biological activity. Overall, the docking analysis underscores that optimal ALKBH5 inhibition by oxindole derivatives relies on the synergistic contribution of three key elements: (i) oxindole core-mediated metal-cofactor mimicry through Asn193/Asp206, (ii) hydrophobic complementarity within the Tyr139/His266/Arg283 pocket, and (iii) specific polar interactions at the rim of the active site facilitated by Ser217 and Thr254.

**FIGURE 14:**
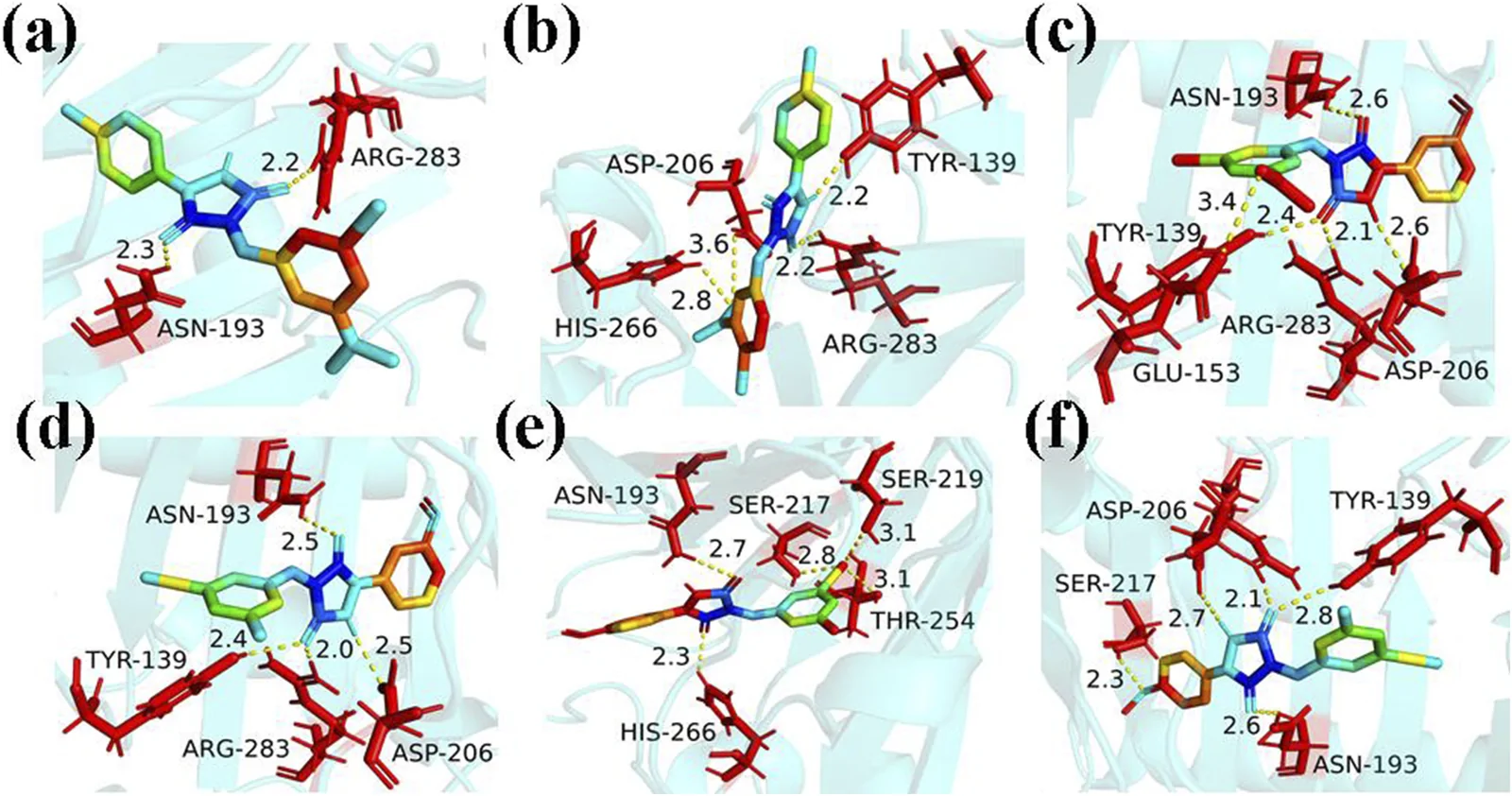
Predicted binding modes of oxindole derivatives within the ALKBH5 active pocket. **(a–f)** Docking poses of representative oxindole analogues in the active site of ALKBH5.

#### 5.2.3 Maleimide derivatives

Multiple maleimide-based compounds were synthesized and pharmacologically profiled as prospective ALKBH5 antagonists, encompassing two structural subclasses: as shown in Figure 15, N-arylethyl maleimides **33–38** and 3,4-diaryl maleimides 39–41 (Supplementary Table S1; Supplementary Table S2) (Liang et al., 2025; Wu et al., 2025). In the N-arylethyl series, biochemical assessment by fluorescence polarization (FP) assay identified compound **36**, bearing a 4-hydroxyphenylethyl substituent on the maleimide nitrogen, as the analogue with an IC_50_ of 1.1 μM against ALKBH5 (Liang et al., 2025; Wu et al., 2025). This activity markedly surpassed its regioisomeric counterparts **34** (2-chloro, IC_50_ = 4.53 μM) and **35** (3-chloro, IC_50_ = 0.89 μM), as well as the 2-methoxy derivative **33** (IC_50_ = 0.62 μM), highlighting the pivotal contribution of the para-hydroxyl group to inhibitory potency (Liang et al., 2025; Wu et al., 2025). The 2-(2-hydroxyethoxy)phenyl analogue **38** displayed moderate activity (IC_50_ = 1.76 μM), whereas the ortho-substituted ester-bearing derivative **37** was the least active within this subset (IC_50_ = 0.79 μM), suggesting that bulky or strongly electron-withdrawing ortho substituents are detrimental to optimal binding (Liang et al., 2025; Wu et al., 2025). In the 3,4-diaryl maleimide series, compound **40** emerged as the strongest inhibitor with an IC_50_ of 2.32 μM, outperforming analogues **39** (IC_50_ = 5.344 μM) and **41** (IC_50_ = 9.57 μM). Collectively, these structure–activity relationships demonstrate that a para-hydroxyl-decorated N-arylethyl side chain and an appropriately substituted 3,4-diaryl maleimide scaffold are favorable structural motifs for achieving potent ALKBH5 inhibition (Liang et al., 2025; Wu et al., 2025).

**FIGURE 15:**
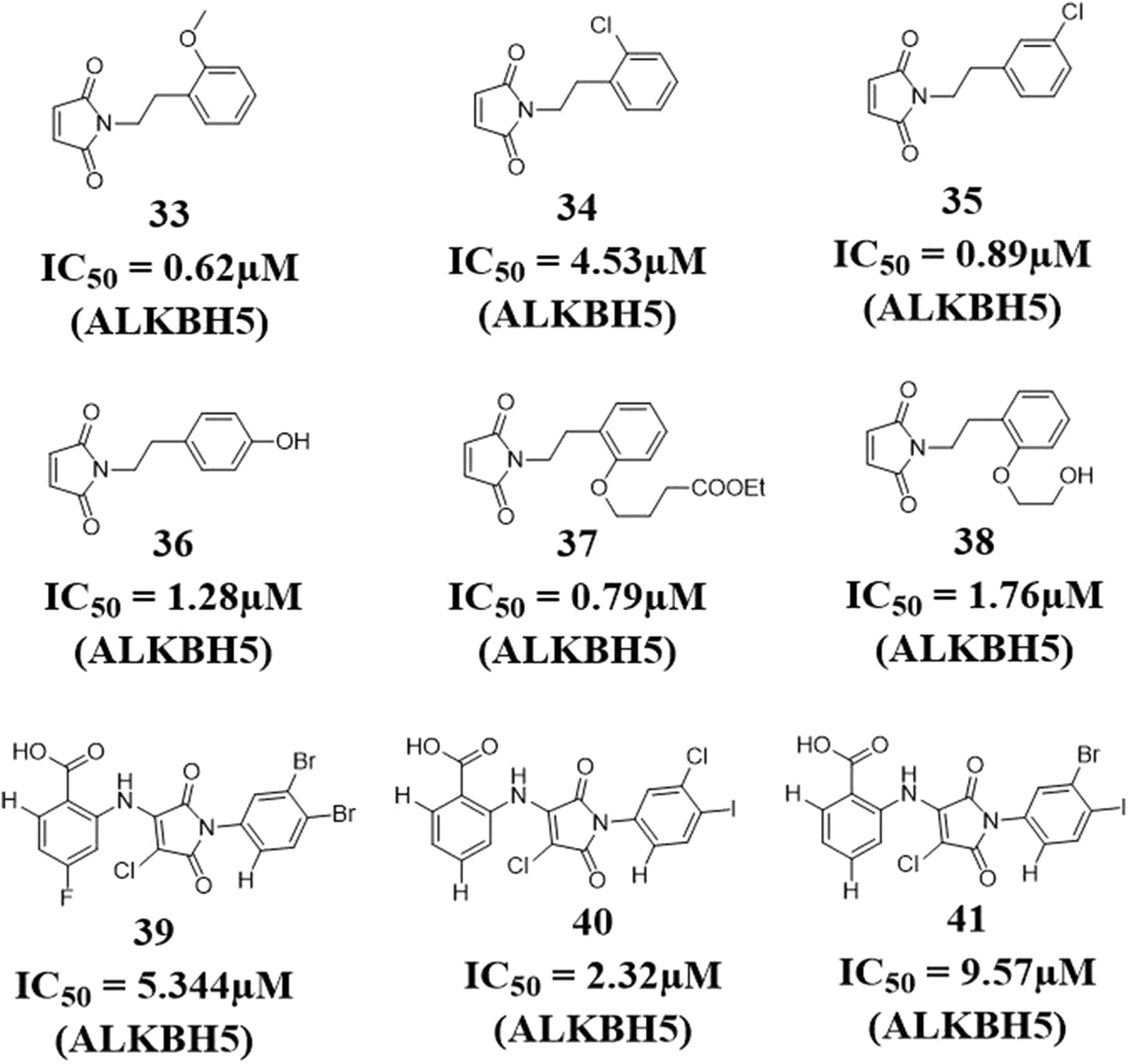
Chemical structures and ALKBH5 inhibitory activities of maleimide derivatives 33–41.

Figure 2a Molecular docking studies were used to rationalize the observed SAR trends and elucidate the binding modes of maleimide derivatives **33–41** within the ALKBH5 catalytic pocket (Figure 16). Across the series, the maleimide carbonyl core was consistently anchored via a conserved hydrogen-bonding network involving Tyr139, Asn193, and Arg283. Notably, the inhibitor **36** (IC_50_ = 1.1 μM) established supplementary polar interactions with Ser217 (2.2 Å) and Arg277 (2.1 Å) mediated by its para-hydroxyl group, thereby reinforcing ligand anchoring beyond the primary maleimide-protein contacts. By contrast, the less active ortho-substituted analogues **33** and **37** engaged only the canonical triad plus a distal contact with His266 (2.8 Å) or Asn193 (2.2 Å), respectively, lacking the additional edge-to-face stabilization afforded by the para-hydroxyl motif. Within the 3,4-diaryl maleimide subset, the superior activity of compound **40** (IC_50_ = 2.32 μM) correlated with its capacity to form dual hydrogen bonds with Arg283 (1.9 and 2.0 Å) alongside interactions with Tyr139 (2.7 Å) and Asn193 (2.3 Å), whereas the weakest analogue **41** (IC_50_ = 9.57 μM) forfeited one of these Arg283 contacts and presented a more restricted interaction network limited to Asn193 (2.7 Å) and Arg283 (2.1 Å). Taken together, the docking analyses reveal that inhibitory potency across the maleimide series is governed by the extent of hydrogen-bonding complementarity with the catalytic pocket, particularly the ability to engage auxiliary residues such as Ser217 and Arg277 in addition to the central Tyr139/Asn193/Arg283 anchor.

**FIGURE 16:**
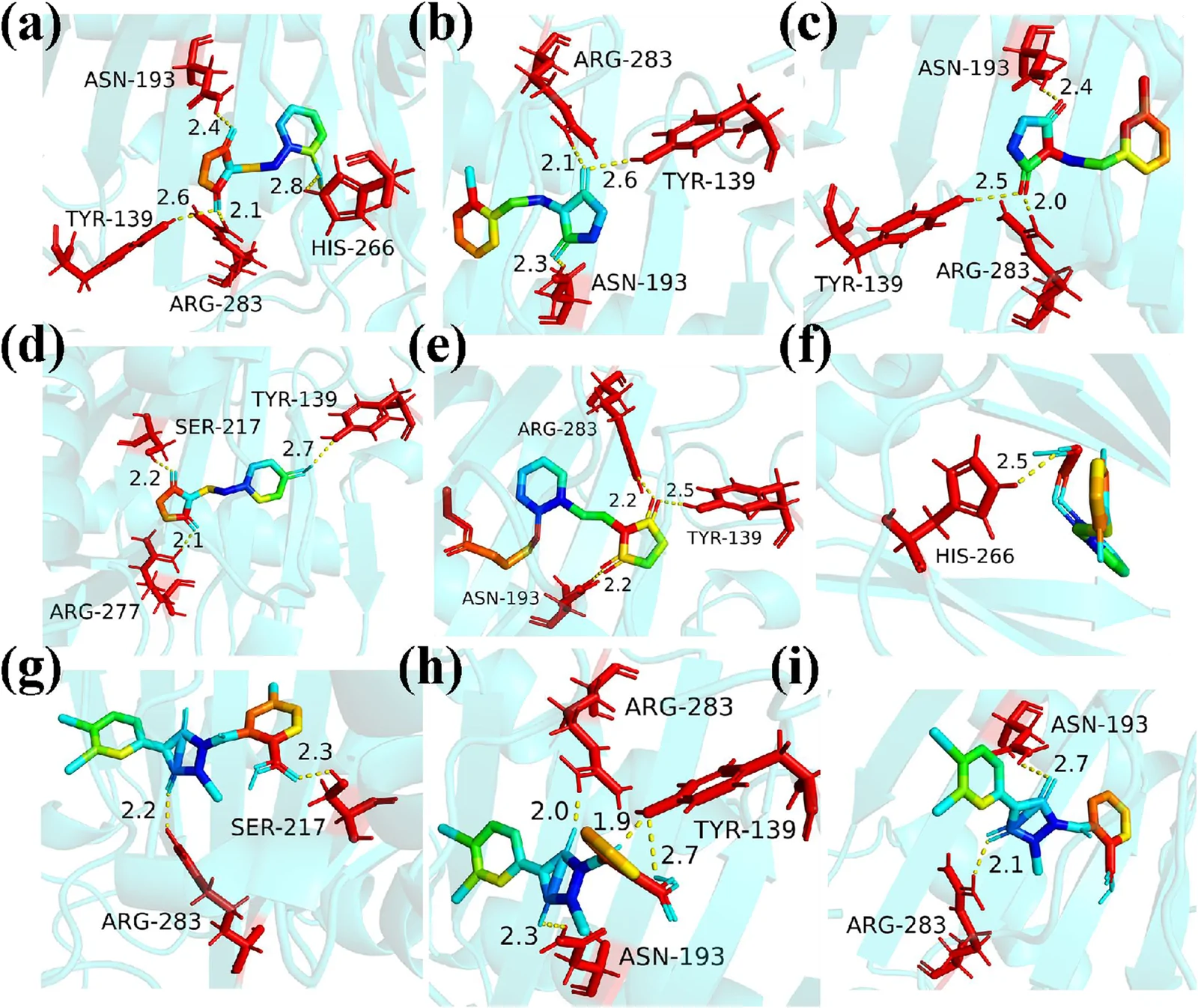
Predicted binding modes of maleimide derivatives 33–41 within the ALKBH5 active pocket (PDB: 4NRO) derived from molecular docking simulations. **(a)** 33, **(b)** 34, **(c)** 35, **(d)** 36, **(e)** 37, **(f)** 38, **(g)** 39, **(h)** 40, **(i)** 41. Amino acid residues critical for ligand recognition are shown as red sticks; putative hydrogen bonds are denoted by yellow dashed lines with distances in angstroms (Å).

## 6 Challenges in the development of ALKBH5 inhibitors

### 6.1 Hurdles of selectivity

As members of the Fe^II^/α-KG-dependent oxygenase family, ALKBH5 and FTO exhibit a shared structural blueprint centered on the canonical DSBH fold. This conserved topology incorporates the functionally essential His-Asp-His triad for metal ion ligation and a dedicated 2-oxoglutarate (2-OG) binding cavity (Aik et al., 2014; Xu et al., 2014). Despite sharing the same superfamily, ALKBH5 possesses a markedly more compact catalytic pocket than its paralog FTO, providing a structural basis for selective orthosteric inhibition (Gao et al., 2024), the high topological similarity between their catalytic pockets remains the principal source of off-target risk. Most reported ALKBH5 inhibitors are either 2-OG analogs or pan-inhibitors that simultaneously target both FTO and ALKBH5, whereas truly selective small molecules are extremely scarce (Gao et al., 2024). Recent progress has demonstrated that covalent strategies targeting ALKBH5-unique residues, such as the maleimide derivative **33** binding Cys200 (Wu et al., 2025) or the salicylaldehyde-based compound **25** forming an imine with Lys132 (Fei et al., 2025) can achieve excellent selectivity over FTO and ALKBH3. Likewise, structure-guided optimization of maleimide derivatives has furnished potent and selective ALKBH5 blockers by engaging non-catalytic cysteine residues (Liang et al., 2025). Nevertheless, how to systematically exploit these subtle structural differences, such as the nucleotide recognition lid (NRL) and the Cys230-Cys267 disulfide bond to finely discriminate ALKBH5 from FTO while retaining potent target engagement remains an unresolved challenge at the interface of structural biology and medicinal chemistry.

### 6.2 Optimization of druggability

The transition from lead compounds to clinical candidates is frequently impeded by suboptimal metabolic stability, limited oral bioavailability, and potential systemic toxicity. For instance, although IOX1 effectively inhibits ALKBH5 and restores global m^6^A levels, its encapsulation within surface-modified bioengineered ferritin nanocages (HSSS) was required to achieve targeted delivery to the infarct region, implicitly indicating unfavorable pharmacokinetic properties of the free compound (Cheng et al., 2022). Similarly, the optimized covalent inhibitor compound **25** exhibits high binding affinity (K_d_ = 2.80 ± 0.55 μM by ITC) and favorable selectivity for ALKBH5 over FTO and ALKBH3 (Fei et al., 2025; Tang et al., 2018). However, the metabolic lability of its aldehyde warhead and the risk of off-target covalent modification necessitate rigorous preclinical safety profiling. It is also noteworthy that ALKBH5 is indispensable for male spermatogenesis and oocyte meiotic maturation (Bai et al., 2023; Tang et al., 2018), implying that chronic systemic inhibition may elicit unanticipated adverse effects such as reproductive toxicity. Consequently, candidate drugs must be equipped with robust tumor-targeting delivery strategies or localized administration routes to widen the therapeutic window, further complicating the druggability optimization landscape.

To safely administer ALKBH5 inhibitors systemically, three parallel risk-mitigation strategies should be pursued. (i) Tumor-targeted delivery systems: As exemplified by the ferritin nanocage encapsulation of IOX1 (HSSS-I) for myocardial infarction (Cheng et al., 2022), nanoparticle-based or antibody-drug conjugate platforms could restrict ALKBH5 inhibition to the tumor microenvironment, minimizing systemic exposure and reproductive toxicity. (ii) PROTAC-mediated targeted degradation: Unlike occupancy-driven inhibitors, PROTACs achieve catalytic target elimination and may allow lower effective doses, reducing off-tissue effects. The established FTO-PROTAC framework (Liu X. et al., 2024) provides a practical blueprint for developing ALKBH5-specific degraders, which could selectively degrade ALKBH5 in tumor cells while sparing normal tissues. (iii) Inducible or localized administration routes: For solid tumors, intratumoral injection or hypoxia-activated prodrugs could confine ALKBH5 inhibition to the tumor niche, avoiding the physiological requirements of ALKBH5 in testis and ovary (Tang et al., 2018).

### 6.3 Mechanisms of resistance

Long-term ALKBH5 inhibition is anticipated to provoke adaptive escape through three convergent mechanisms. First, target-dependent alterations. Tumor cells may acquire resistance mutations at the catalytic site or covalent-target residues (Lys132, Cys200) that weaken inhibitor binding. More importantly, post-translational modifications function as adaptive molecular switches: USP36-mediated deubiquitination stabilizes ALKBH5 protein to compensate for pharmacological blockade, whereas KAT8-driven acetylation and EGFR-activated phosphorylation dynamically modulate enzymatic activity and substrate specificity (Chang et al., 2023; Yu F. et al., 2021; Zhang et al., 2023). Second, epitranscriptomic bypass and metabolic reprogramming. Cancer cells can counteract by elevating the paralogue demethylase FTO or rewiring methyltransferase complexes to maintain m^6^A homeostasis (Qu et al., 2022). Additionally, tumors may circumvent ALKBH5 dependency by activating compensatory metabolic routes—such as shifting from ALKBH5-regulated GLUT4-mediated glycolysis to alternative energy pathways—to sustain proliferation and therapeutic evasion (Liu H. et al., 2022). Third, microenvironment-driven heterogeneity and immune evasion. Intratumoral hypoxia transcriptionally upregulates FTO, which elevates PD-L1 and blunts T-cell attack, creating an immune-evasive niche that persists despite ALKBH5 suppression (Hua et al., 2024). In glioblastoma, radiation-responsive activation of the MST4-USP14 axis stabilizes ALKBH5 and enhances DNA repair, indicating that DNA-damage therapies may inadvertently select for resistant clones (Zhou et al., 2025). Collectively, these multi-layered mechanisms underscore that monotherapy will likely face rapid adaptive escape, necessitating combination strategies that concurrently neutralize compensatory nodes.

## 7 Future prospects for the development of ALKBH5 inhibitors

### 7.1 Development of novel inhibitors

The continuous elucidation of ALKBH5 crystal structures has furnished atomic-level templates for structure-guided inhibitor design. The evolutionarily preserved double-stranded *β*-helix (DSBH) topology is complemented by distinctive structural motifs, including the nucleotide-binding lid and a cysteine bridge connecting Cys230 and Cys267, provides druggable pockets that can be exploited to discriminate ALKBH5 from its paralog FTO (Aik et al., 2014). Recent successes in integrating fluorescence polarization-based high-throughput screening with rational SAR optimization, exemplified by the development of 1-aryl-1H-pyrazole derivatives and pyrazolo [1,5-a]pyrimidine scaffolds, demonstrate that iterative medicinal chemistry campaigns can deliver sub-micromolar, selective ALKBH5 inhibitors (Li W. et al., 2023; Shen et al., 2020). Moreover, the emergence of covalent inhibitors targeting non-catalytic cysteine or lysine residues has opened new chemical space for achieving exquisite selectivity (Feng et al., 2014; Wang Y. et al., 2023; Yang et al., 2025). Looking forward, artificial intelligence-driven generative models and deep-learning-based affinity prediction are expected to accelerate hit-to-lead optimization beyond traditional SAR exploration. The development of bifunctional degraders such as PROTACs, as well as dual-target inhibitors simultaneously engaging ALKBH5 and complementary epigenetic nodes, represents a promising next-generation strategy that may overcome the limitations of conventional occupancy-driven inhibition.

### 7.2 Combination therapeutic strategies

Given the pleiotropic role of ALKBH5 in driving therapy resistance, monotherapy with ALKBH5 inhibitors may face rapid adaptive escape; therefore, rational combination regimens are essential. Preclinical evidence strongly supports co-administration with cytotoxic chemotherapy: ALKBH5 inhibition destabilizes oncogenic transcripts such as TACC3 and ITPA in acute myeloid leukemia (Xu et al., 2014), restores sensitivity to paclitaxel in non-small cell lung cancer (Gao et al., 2024), enhances gemcitabine efficacy in pancreatic cancer (Zhou et al., 2025), overcomes cisplatin resistance in gastric and epithelial ovarian cancers (Nie et al., 2021b; Zhang et al., 2022), and resensitizes triple-negative breast cancer to doxorubicin. Furthermore, ALKBH5-mediated radioresistance in glioblastoma and hepatocellular carcinoma indicates that ALKBH5 blockade may function as an effective radiosensitizer when combined with ionizing radiation (Rivara et al., 2024). Notably, ALKBH5 regulates PD-L1 expression and immune evasion across multiple malignancies, including non-small cell lung cancer, intrahepatic cholangiocarcinoma, and glioma (Rivara et al., 2024), and modulates the tumor immune microenvironment to influence anti-PD-1 therapy response. Consequently, combining ALKBH5 inhibitors with immune checkpoint blockade holds substantial potential to convert immunologically “cold” tumors into “hot” ones, thereby achieving durable antitumor immunity.

### 7.3 Clinical translation

Despite robust preclinical efficacy, no ALKBH5-targeted agent has yet entered human clinical trials, underscoring the urgent need to bridge the gap between bench and bedside. The clinical development trajectory of epigenetic demethylase inhibitors offers valuable guidance. Given the context-dependent dichotomy of ALKBH5 function—oncogenic in acute myeloid leukemia, glioblastoma, and breast cancer, yet tumor-suppressive in hepatocellular carcinoma and pancreatic cancer (Table 1)—blanket systemic inhibition carries inherent risks of paradoxically promoting tumor growth in specific malignancies. To mitigate this, we propose a biomarker-guided precision enrollment strategy analogous to HER2-targeted therapy or PD-L1 companion diagnostics. Specifically, ALKBH5 expression levels and m^6^A landscape signatures should serve as predictive biomarkers for patient stratification. Tumors with confirmed ALKBH5 overexpression and concomitant m^6^A hypomethylation (e.g., AML with elevated *TACC3/AXL/ITPA*, glioblastoma with *NANOG/FOXM1* upregulation) represent the primary target population (Li W. et al., 2023; Li et al., 2020; Zhang et al., 2017). Conversely, malignancies where ALKBH5 acts as a tumor suppressor (e.g., HCC with *PAQR4* destabilization, pancreatic cancer with *PER1* activation) should be excluded from monotherapy trials (Chen et al., 2020; Guo et al., 2020). Furthermore, dynamic m^6^A monitoring during treatment could function as a pharmacodynamic biomarker, ensuring on-target engagement without exceeding thresholds that would disrupt physiological spermatogenesis or oocyte maturation (Tang et al., 2018). For instance, the LSD1 inhibitor LH-1802, discovered through structure-based optimization, has successfully advanced into Phase I clinical trials for relapsed or refractory acute myeloid leukemia and myelodysplastic syndromes, demonstrating that targeted demethylase inhibitors can achieve acceptable safety profiles in hematologic indications (Gao et al., 2024). This precedent suggests that ALKBH5 inhibitors should initially prioritize hematologic malignancies, where ALKBH5 oncogenic dependency is well established and patient stratification via biomarker-driven enrichment is most feasible, before expanding into solid tumor indications. Moreover, given the indispensable physiological roles of ALKBH5 in male spermatogenesis and oocyte meiotic maturation (Liang et al., 2025; Rivara et al., 2024), early-phase clinical trials must incorporate rigorous reproductive toxicity monitoring, pharmacokinetic–pharmacodynamic modeling, and dose-escalation designs to ensure an adequate therapeutic index. Ultimately, the establishment of standardized m^6^A biomarkers and the execution of well-designed Phase I/II studies will be pivotal in realizing the clinical potential of ALKBH5-directed epitranscriptomic therapy.

### 7.4 Comparative therapeutic landscape: ALKBH5 versus METTL3 inhibition

It is instructive to contextualize ALKBH5-directed “eraser” inhibition against parallel efforts targeting METTL3, the catalytic “writer” of m^6^A. METTL3 is frequently overexpressed in AML, where it drives leukemogenesis by stabilizing oncogenic transcripts (e.g., *MYC, BCL2, MDM2*) (Prajapati et al., 2026). Three classes of METTL3 modulators have emerged: substrate-competitive inhibitors (e.g., STM2457, UZH2), allosteric inhibitors (e.g., CDIBA, Eltrombopag), and PROTAC degraders (e.g., STC-15, KH12) (Prajapati et al., 2026).

These two epitranscriptomic strategies face distinct challenges. METTL3 competitive inhibitors target the conserved S-adenosylmethionine (SAM)-binding pocket, risking cross-reactivity with other methyltransferases (Prajapati et al., 2026), whereas recent ALKBH5 covalent inhibitors (e.g., compounds 25, 33) exploit unique non-catalytic residues (Lys132, Cys200) and a more compact active site, achieving superior selectivity over FTO (Fei et al., 2025; Wu et al., 2025). Conversely, both METTL3 and ALKBH5 inhibitors may disrupt normal physiological functions—METTL3 inhibition impairs erythropoiesis (Prajapati et al., 2026), while ALKBH5 loss affects spermatogenesis and oocyte maturation (Prajapati et al., 2026; Tang et al., 2018)—necessitating careful dosing. Resistance mechanisms also converge: compensatory upregulation of FTO or METTL14 may respectively bypass ALKBH5 or METTL3 blockade (Chang et al., 2023; Qu et al., 2022).

Clinically, METTL3-targeted agents have advanced further: STC-15 has entered Phase I trials, and STM2457 shows robust anti-leukemia efficacy (Prajapati et al., 2026; Yankova et al., 2021). In contrast, no ALKBH5 inhibitor has yet reached clinical evaluation. However, ALKBH5 inhibitors offer unique advantages in targeting cancer stem cell self-renewal, immune evasion (via PD-L1 regulation), and glioblastoma stemness (Qiu et al., 2021; Shen et al., 2020; Yankova et al., 2021). Thus, “writer” and “eraser” inhibition represent complementary pillars of m^6^A-targeted oncology, and lessons from the METTL3 clinical trajectory—biomarker-driven stratification and combination with chemotherapy or venetoclax—should inform future ALKBH5 drug development.

## 8 Conclusion

This review systematically summarizes the research advances of ALKBH5 as an m^6^A demethylase in tumor epitranscriptomic regulation and the current status of small-molecule inhibitor development, elucidating its core value as a precision therapeutic target in the RNA epigenetic landscape. ALKBH5 exerts oncogenic driving effects in acute myeloid leukemia, glioblastoma, and breast cancer by demethylating and stabilizing target mRNAs such as *FOXM1*, and *NANOG*, in an opposing pathological setting, the enzyme operates as a growth-inhibitory factor in both hepatocellular carcinoma and pancreatic neoplasms. Leveraging unique architectural features revealed by ALKBH5 crystal structures, including the double-stranded β-helix (DSBH) fold, the nucleotide recognition lid, and the Cys230-Cys267 disulfide bond, researchers have successfully developed multiple potent inhibitors: non-covalent chemotypes such as 1-aryl-1H-pyrazole derivatives (IC_50_ = 0.021 μM), pyrazolo [1,5-a]pyrimidine scaffolds, and IOX1, and covalent inhibitors achieving exquisite selectivity by targeting Cys200 or Lys132, as exemplified by the maleimide derivative compound **33**, the salicylaldehyde-based compound **25** (FP IC_50_ = 0.49 μM), and oxindole derivatives. Nevertheless, critical bottlenecks constraining clinical translation persist, including off-target risks arising from the high structural homology between ALKBH5 and FTO, the metabolic lability of certain lead compounds and potential reproductive toxicity, and adaptive resistance mechanisms involving USP36-mediated deubiquitinative upregulation or compensatory m^6^A pathway rewiring. Looking forward, artificial intelligence-assisted drug design, degradationproteolysis-targeting chimera (PROTAC)-mediated targeted degradation, ALKBH5/ALKBH2 dual-target inhibitors, and combination strategies with chemotherapy, radiotherapy, or immune checkpoint blockade are expected to offer novel avenues for overcoming drug resistance and widening the therapeutic window. Drawing on the successful advancement of the LSD1 inhibitor LH-1802 into Phase I clinical trials, ALKBH5 inhibitors should prioritize biomarker-guided clinical validation in hematologic malignancies, accompanied by the establishment of standardized m^6^A dynamic monitoring systems. In conclusion, the in-depth development of ALKBH5 inhibitors not only holds promise for delivering precision epitranscriptome-targeted therapeutic regimens for malignant tumors but also provides indispensable chemical probes for dissecting the biological functions of ALKBH5, carrying significant scientific importance and broad clinical application prospects. Crucially, the clinical application of ALKBH5 inhibitors must transcend a ‘one-size-fits-all’ paradigm. Rigorous patient stratification based on ALKBH5 expression status, tumor-specific m^6^A signatures, and integrated biomarker monitoring—coupled with advanced drug delivery technologies—will be indispensable to harness the therapeutic potential of ALKBH5 inhibition while averting context-dependent adverse outcomes, including the inadvertent promotion of tumor growth in ALKBH5 tumor-suppressive malignancies.
