# Supplementary material for: ALKBH5 inhibitors for cancer therapy: molecular docking, challenges and future prospects
Source: Front Pharmacol. 2026 Sep 25;17:1946973. doi: 10.3389/fphar.2026.1946973 (PMC13650563; doi:10.3389/fphar.2026.1946973)
Supplement: Supplementary file 1 [file DataSheet1.docx]

**Table S1. Chemical Structure and Enzyme Inhibition Activity Comparison of ALKBH5 Inhibitors**

| **Compound Name** | **Structure** | **IC_50_ (µM)** | **Reference** |
| --- | --- | --- | --- |
| **1 (5)** |  | **4.253** | **(Fang et al., 2022)** |
| **2 (20v)** |  | **0.061** |  |
| **3 (20m)** |  | **0.021** |  |
| **4 (20w)** |  | **0.396** |  |
| **5 (20t)** |  | **0.421** |  |
| **6 (20u)** |  | **0.559** |  |
| **7 (20d)** |  | **0.591** |  |
| **8 (20l)** |  | **0.727** |  |
| **9 (20s)** |  | **0.89** |  |
| **10 (20c)** |  | **2.734** |  |
| **11 (20ab)** |  | **10.23** |  |
| **12 (20n)** |  | **4.944** |  |
| **13 (20ac)** |  | **5.038** |  |
| **14 (20aa)** |  | **6.607** |  |
| **15 (20j)** |  | **6.615** |  |
| **16 (8539-0746)** |  | **18.59** | **(Wang et al., 2023)** |
| **17 (6)** |  | **14.44** |  |
| **18 (14)** |  | **9.12** |  |
| **19 (15)** |  | **7.88** |  |
| **20 (18^b^)** |  | **4.64** |  |
| **21 (19^b^)** |  | **2.97** |  |
| **Ena15** |  | **18.3** | **(Takahashi et al., 2022)** |
| **Ena21** |  | **15.7** |  |
| **22 (6)** |  | **25** | **(Rivara et al., 2024)** |
| **IOX1** |  | **-** | **(Rivara et al., 2024)** |
| **DDO-2728** |  | **4.06** | **(Fei et al., 2025)** |
| **23 (17)** |  | **7.44** |  |
| **24 (21)** |  | **3.40** |  |
| **25 (23)** |  | **1.43** |  |
| **26 (24)** |  | **8.56** |  |
| **27 (W23-1001)** |  | **5.027** | **(Yang et al., 2025)** |
| **28**  **(W23-1004)** |  | **8.532** |  |
| **29 (W23-1005)** |  | **5.608** |  |
| **30**  **(W23-1006)** |  | **3.848** |  |
| **31**  **(W23-1007)** |  | **6.126** |  |
| **32**  **(W23-1008)** |  | **8.663** |  |
| **33 (18l)** |  | **0.62** | **(Wu et al., 2025)** |
| **34 (18n)** |  | **4.53** |  |
| **35 (18o)** |  | **0.89** |  |
| **36 (18p)** |  | **1.28** |  |
| **37 (30i)** |  | **0.79** |  |
| **38 (32)** |  | **1.76** |  |
| **39 (4)** |  | **5.344** | **(Liang et al., 2025)** |
| **40 (18)** |  | **2.32** |  |
| **41 (27)** |  | **9.57** |  |

**Table S2. Binding Affinity, Key Interaction Residues, Selectivity, and Development Stage of ALKBH5 Inhibitors**

| **Compound Name** | **Binding Affinity (kCal/mol)** | **Binding Mode** | **Key Interacting Residues** | **Selectivity profile** | **Development stage** | **Reference** |
| --- | --- | --- | --- | --- | --- | --- |
| **1 (5)** | **-7.312** | **non-covalent docking** | **Asp206, Ser217, Lys132, Ser219** | **-** | **Biochemical assay; compound 3: In vitro (cellular)** | **(Fang et al., 2022)** |
| **2 (20v)** | **-7.017** | **non-covalent docking** | **Arg277** | **-** | **Biochemical assay; compound 3: In vitro (cellular)** |  |
| **3 (20m)** | **-7.71** | **non-covalent docking** | **Arg277， Asp206，His266** | **high selectivity, showing negligible activity against the paralogue FTO and other AlkB subfamily members (ALKBH1, ALKBH2)** | **Biochemical assay; compound 3: In vitro (cellular)** |  |
| **4 (20w)** | **-7.103** | **non-covalent docking** | **Asp206，Lys132，Asn193** | **-** | **Biochemical assay; compound 3: In vitro (cellular)** |  |
| **5 (20t)** | **-7.544** | **non-covalent docking** | **Arg277, Asp206, Asn193** | **-** | **Biochemical assay; compound 3: In vitro (cellular)** |  |
| **6 (20u)** | **-7.143** | **non-covalent docking** | **Arg277, Ser219** | **-** | **Biochemical assay; compound 3: In vitro (cellular)** |  |
| **7 (20d)** | **-7.134** | **non-covalent docking** | **Arg277, Asp206, Asn193** | **-** | **Biochemical assay; compound 3: In vitro (cellular)** |  |
| **8 (20l)** | **-7.268** | **non-covalent docking** | **Asp206，Lys132，Asn193** | **-** | **Biochemical assay; compound 3: In vitro (cellular)** |  |
| **9 (20s)** | **-7.913** | **non-covalent docking** | **Lys132, Asn193, Ser219, Ser217, Arg277** | **-** | **Biochemical assay; compound 3: In vitro (cellular)** |  |
| **10 (20c)** | **-7.668** | **non-covalent docking** | **Asn193** | **-** | **Biochemical assay; compound 3: In vitro (cellular)** |  |
| **11 (20ab)** | **-8.315** | **non-covalent docking** | **Arg277, His266, and Lys132** | **-** | **Biochemical assay; compound 3: In vitro (cellular)** |  |
| **12 (20n)** | **-8.412** | **non-covalent docking** | **Arg277, His266, and Lys132** | **-** | **Biochemical assay; compound 3: In vitro (cellular)** |  |
| **13 (20ac)** | **-8.458** | **non-covalent docking** | **Lys132, Try139,**  **His204, Arg277, Asp206** | **-** | **Biochemical assay; compound 3: In vitro (cellular)** |  |
| **14 (20aa)** | **-7.284** | **non-covalent docking** | **Lys132, Arg277,**  **Ser217,**  **Ser219,**  **Arg283** | **-** | **Biochemical assay; compound 3: In vitro (cellular)** |  |
| **15 (20j)** | **-7.67** | **non-covalent docking** | **Lys132,**  **Arg283** | **-** | **Biochemical assay; compound 3: In vitro (cellular)** |  |
| **16 (8539-0746)** | **-9.114** | **non-covalent docking** | **Arg130,**  **Asp206** | **-** | **Biochemical assay; compound 21: In vitro (cellular)** | **(Wang et al., 2023)** |
| **17 (6)** | **-9.252** | **non-covalent docking** | **Asp206,**  **Arg130,**  **Val202** | **-** | **Biochemical assay; compound 21: In vitro (cellular)** |  |
| **18 (14)** | **-9.651** | **non-covalent docking** | **Asp206,**  **Arg130,**  **Arg277** | **-** | **Biochemical assay; compound 21: In vitro (cellular)** |  |
| **19 (15)** | **-8.962** | **non-covalent docking** | **Asp125,**  **Arg126,**  **Val124,**  **Glu121,**  **Tyr139,**  **His122** | **-** | **Biochemical assay; compound 21: In vitro (cellular)** |  |
| **20 (18^b^)** | **-9.185** | **non-covalent docking** | **Arg126,**  **Tyr139,**  **His122** | **-** | **Biochemical assay; compound 21: In vitro (cellular)** |  |
| **21 (19^b^)** | **-9.392** | **non-covalent docking** | **Asp125** | **-** | **Biochemical assay; compound 21: In vitro (cellular)** |  |
| **Ena15** | **-7.546** | **non-covalent docking** | **Tyr139,**  **Arg130** | **Selective for ALKBH5 over FTO (enhances FTO activity at 80 μM)** | **Biochemical assay** | **(Takahashi et al., 2022)** |
| **Ena21** | **-7.342** | **non-covalent docking** | **Arg277** | **Selective for ALKBH5 over FTO (36% inh. at 80 μM)** | **Biochemical assay** |  |
| **22 (6)** | **-6.93** | **non-covalent docking** | **Arg283** | **Dual ALKBH2/ALKBH5 inhibitor** | **In vitro (enzyme, cellular)** | **(Rivara et al., 2024)** |
| **IOX1** | **-** | **non-covalent docking** | **-** | **-** | **In vitro (cellular) , in vivo (rat AMI model)** | **(Rivara et al., 2024)** |
| **DDO-2728** | **-9.122** | **covalent docking** | **Tyr156,**  **Lys132** | **-** | **Biochemical assay** | **(Fei et al., 2025)** |
| **23 (17)** | **-8.383** | **covalent docking** | **Lys132,**  **Tyr139,**  **Asp206,**  **Asn193** | **-** | **Biochemical assay** |  |
| **24 (21)** | **-8.146** | **covalent docking** | **Lys132,**  **Tyr139,**  **Asp206,**  **Asn193,**  **Arg283,**  **Arg277** | **-** | **Biochemical assay** |  |
| **25 (23)** | **-8.532** | **covalent docking** | **Tyr139,**  **His204,**  **His266,**  **Asn193,**  **Arg283,**  **Tyr195** | **-** | **In vitro (enzyme, cellular)** |  |
| **26 (24)** | **-8.565** | **covalent docking** | **Lys132, Tyr139,**  **Arg283** | **-** | **Biochemical assay** |  |
| **27 (W23-1001)** | **-8.524** | **covalent docking** | **Arg283,**  **Asn193** | **-** | **Biochemical assay** | **(Yang et al., 2025)** |
| **28**  **(W23-1004)** | **-8.519** | **covalent docking** | **Asp206,**  **His266,**  **Tyr139,**  **Arg283** | **-** | **Biochemical assay** |  |
| **29 (W23-1005)** | **-8.793** | **covalent docking** | **Asp206,**  **Glu153,**  **Tyr139,**  **Arg283,**  **Asn193** | **-** | **Biochemical assay** |  |
| **30**  **(W23-1006)** | **-8.431** | **covalent docking** | **Asn193,**  **Tyr139,**  **Arg283,**  **Asp206** | **-** | **Biochemical assay** |  |
| **31**  **(W23-1007)** | **-8.733** | **covalent docking** | **Asn193,**  **Ser217,**  **Ser219,**  **Thr254,**  **His266** | **-** | **Biochemical assay** |  |
| **32**  **(W23-1008)** | **-7.956** | **covalent docking** | **Asp206,**  **Ser217,**  **Asn193,**  **Tyr139** | **-** | **Biochemical assay** |  |
| **33 (18l)** | **-6.767** | **covalent docking** | **Asn193,**  **Arg283,**  **Tyr139,**  **His266** | **Selective over FTO and ALKBH3** | **In vitro (cellular), in vivo** | **(Wu et al., 2025)** |
| **34 (18n)** | **-6.812** | **covalent docking** | **Asn193,**  **Arg283,**  **Tyr139** | **-** | **Biochemical assay** |  |
| **35 (18o)** | **-6.656** | **covalent docking** | **Asn193,**  **Arg283,**  **Tyr139** | **-** | **Biochemical assay** |  |
| **36 (18p)** | **-6.782** | **covalent docking** | **Tyr139,**  **Ser217,**  **Arg277** | - | **Biochemical assay** |  |
| **37 (30i)** | **-6.7** | **covalent docking** | **Asn193,**  **Arg283,**  **Tyr139** | **-** | **Biochemical assay** |  |
| **38 (32)** | **-6.697** | **covalent docking** | **His266** | **-** | **Biochemical assay** |  |
| **39 (4)** | **-8.575** | **covalent docking** | **Ser217,**  **Arg283** | **-** | **Biochemical assay** | **(Liang et al., 2025)** |
| **40 (18)** | **-7.799** | **covalent docking** | **Tyr139,**  **Asn193,**  **Arg283** | - | **Biochemical assay** |  |
| **41 (27)** | **-8.433** | **covalent docking** | **Asn193,**  **Arg283** | **-** | **Biochemical assay** |  |

**Reference**

Fang, Z., Mu, B., Liu, Y., Guo, N., Xiong, L., Guo, Y., Xia, A., Zhang, R., Zhang, H., Yao, R., Fan, Y., Li, L., Yang, S., Xiang, R., 2022. Discovery of a potent, selective and cell active inhibitor of m6A demethylase ALKBH5. European Journal of Medicinal Chemistry 238, 114446.

Fei, W.-L., Wang, Y.-Z., Feng, Q.-L., Li, C.-T., Jiang, R.-X., Zhang, S.-D., Pan, Y., Ni, P.-F., Huang, F.-R., Huangfu, Y.-B., Zhang, S.-D., You, Q.-D., Guo, X.-K., 2025. Discovery of the salicylaldehyde-based compound DDO-02267 as a lysine-targeting covalent inhibitor of ALKBH5. European journal of medicinal chemistry 284, 117183.

Liang, L., Fei, W., Wang, Y., Zhang, Z., You, Q., Guo, X., 2025. Discovery of maleimide derivatives as m6A demethylase ALKBH5 inhibitors. Bioorganic & Medicinal Chemistry 120, 118083.

Rivara, M., Nicolini, G., Malacrida, A., Re, F., Incerti, M., Russo, G., Zuliani, V., 2024. New ALKBH2 and ALKBH5 inhibitors for treating glioblastoma. Results in Chemistry 9, 101645.

Takahashi, H., Hase, H., Yoshida, T., Tashiro, J., Hirade, Y., Kitae, K., Tsujikawa, K., 2022. Discovery of two novel ALKBH5 selective inhibitors that exhibit uncompetitive or competitive type and suppress the growth activity of glioblastoma multiforme. Chem Biol Drug Des 100(1), 1–12.

Wang, Y.-Z., Li, H.-Y., Zhang, Y., Jiang, R.-X., Xu, J., Gu, J., Jiang, Z., Jiang, Z.-Y., You, Q.-D., Guo, X.-K., 2023. Discovery of Pyrazolo[1,5- a ]pyrimidine Derivative as a Novel and Selective ALKBH5 Inhibitor for the Treatment of AML. Journal of medicinal chemistry 66(23), 15944–15959.

Wu, H., Lai, G.-Q., Cheng, R., Huang, H., Wang, J., Liu, Z., Gao, J., Zhou, H., Li, C., Yang, C.-G., Liu, H., 2025. Discovery of Covalent and Cell-Active ALKBH5 Inhibitors with Potent Antileukemia Effects In Vivo. Angewandte Chemie International Edition 64(18), e202424928.

Yang, X., Huang, K., Wu, X.-N., Zhang, C., Sun, Y., Gao, Y., Zhou, J., Tao, L., Zhang, H., Wu, Y., Luo, H.-B., Wang, H., 2025. Discovery of a Novel Selective and Cell-Active N6‑Methyladenosine RNA Demethylase ALKBH5 Inhibitor. Journal of Medicinal Chemistry 68(4), 4133–4147.
